# Two universal pathways in demographic transition

**DOI:** 10.1017/ehs.2026.10054

**Published:** 2026-06-15

**Authors:** Kenji Itao

**Affiliations:** 1 Frontier Research Institute for Interdisciplinary Sciences, Tohoku University, Aramaki aza Aoba 6-3, Aoba-ku, Sendai, Japan; 2 Center for Brain Science, RIKEN, 2-1 Hirosawa, Wako, Saitama, Japan

**Keywords:** demographic transition, fertility decline, global data analysis, Global data reveal two universal pathways linking fertility and longevity countries, follow or switch between them

## Abstract

Demographic transition, characterised by declines in fertility and mortality, is a global phenomenon associated with modernisation. While typical patterns of fertility decline have been described mainly in Western countries, their applicability to other regions and the underlying mechanisms remain unclear. Using data spanning 1800–2020 from 237 countries and territories, this study identifies two universal pathways in the change in the crude birth rate (births per 1,000 person-years, λ) and period life expectancy at birth (*e*_0_). Most countries’ demographic trajectories follow one of these two pathways or shift between them. These pathways define phases governed by different mechanisms. Phase I, conserving λ*e*_0_, dominated until the mid-20th century and was characterised by high child mortality and steady population growth. By contrast, Phase II, conserving λexp(*e*_0_/17), has prevailed since 1950 and is marked by low child mortality and steady growth in GDP per capita. A theoretical model considering the trade-off between reproduction and education elucidates the transition between these phases. The transition to Phase II is accelerated by declining educational costs, rising social mobility, and cultural transmission linked to modernisation and Westernisation. This study demonstrates quantitative regularities between fertility and longevity during demographic transition and provides a theoretical lens for their underlying mechanisms.

## Graphical abstract

**Figure:**
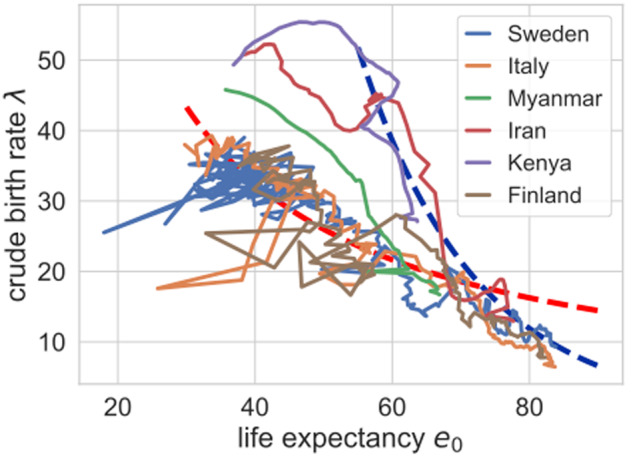

## 1. Introduction

Declines in fertility and mortality, commonly referred to as the demographic transition, are widely observed in modern societies (Kirk, 1996; Notestein, 1945). Modernisation typically initiates this transition with a decline in mortality, leading to rapid population growth, followed by a later decrease in fertility and, more recently, population shrinkage (Bongaarts, 2009; Kirk, 1996; Reher, 2012). The economic, social, and political implications of this transition have been examined in demography, economics, and history, while also drawing considerable attention from policymakers and the public alike (Bloom et al., 2003; Murphy, 2017; Reher, 2012). Here, I ask whether demographic transitions across diverse societies share common quantitative regularities, and what mechanisms generate them.

Researchers have long explored the typical patterns of demographic transition. Demographers have identified two stages of demographic transition in Western countries. The initial decline in fertility to the replacement level (i.e., two children per woman) is termed the first demographic transition. By contrast, subsequent declines below the replacement level are referred to as the second demographic transition (Lesthaeghe, 2010, 2014; Zaidi & Morgan, 2017). The shift to the second demographic transition is associated with lower marriage rates, delayed childbearing, the pursuit of ‘higher-order needs’ such as education and self-realisation, and increased female economic empowerment, among other factors (Bongaarts, 2009; Kirk, 1996; Lesthaeghe, 2014; Lutz et al., 2019; Mace, 2000; Mulder, 1998; Zaidi & Morgan, 2017). Similarly, the ‘unified growth theory’ proposes that modern technological advancements drive the transition from the Malthusian growth phase, characterised by stable population growth, to the modern growth phase, marked by constant GDP per capita growth (Galor, 2011). These frameworks suggest the potential universality of demographic dynamics across countries. However, the extent to which such universality applies globally remains an open question, necessitating a comprehensive quantitative investigation (Lesthaeghe, 2014).

The mechanisms driving demographic transitions are multifactorial; although various disciplines have proposed explanations, the fundamental drivers remain elusive. The decline in mortality is generally attributed to improvements in nutrition, infrastructure, and medicine (Canning, 2011; Kirk, 1996). In contrast, explanations for declining fertility span multiple perspectives. Demographers highlight population pressure (Malthus, 1798), cultural shifts towards postmodern norms (Ihara & Feldman, 2004; Lesthaeghe, 2010, 2014; Zaidi & Morgan, 2017), the implementation of family planning programmes (Amin et al., 2002; Murphy, 2017), and human capital accumulation through education (Canning, 2011; Galor, 2011). Economists emphasise how individuals optimise fertility decisions by balancing reproduction and educational investment, commonly referred to as the quantity–quality trade-off (Becker et al., 2010; Bleakley and Lange, 2009; Fernihough, 2017; Hanushek, 1992; Werding, 2014), and by weighing career advancement and leisure preferences against childbearing (Galindev, 2011; Hakim, 2003; Vitali et al., 2009). Human behavioural ecologists, meanwhile, focus on maximising children’s reproductive value through investments in embodied capital, primarily via education (Colleran, 2016; Kaplan, 1996; Lawson & Mace, 2011; Mace, 2000; Sear, 2015; Shenk, 2009). Comparative studies indicate that childbearing in urban societies is costlier, whereas the returns to educational investment are higher than in traditional settings (Colleran et al., 2015; Kaplan, 1996; Mace, 2008). Finally, cultural evolutionists propose that although the preference for smaller family sizes may be maladaptive, it is widely transmitted due to social learning biases and group-level cultures (Boyd, 2008; Colleran, 2016; Ihara & Feldman, 2004; Mulder, 1998; Newson et al., 2007; Richerson & Sear, 2015). These explanations can be broadly categorised into three: (i) population pressure, (ii) the trade-off between education and fertility, and (iii) the cultural transmission of behavioural strategies. However, the contributions of each are only understood in specific cases, and a unifying explanation remains unknown.

To uncover universal patterns and clarify the primary mechanisms of demographic transition, I focus on macroscopic regularities in the relationship between the crude birth rate λ (births per 1,000 individuals per year) and the period life expectancy at birth $e_{0}$ (life-table expectation of life under a given calendar-year mortality schedule). If fertility decline is driven primarily by population pressure, population size is constrained by limited resources. As a population approaches its carrying capacity, per-capita resources decline, reducing fertility and/or increasing mortality (Malthus, 1798). Gradual improvements in productivity or infrastructure can lift the capacity, and population size tracks a slowly expanding capacity. Under the idealised conditions of a stable population with constant age-specific fertility and mortality rates and zero net migration (Preston et al., 2000, p. 141), $(\lambda e_{0}/1000-1)/e_{0}$ provides a rough measure of the growth rate (Schindler et al., 2012). Hence, $\lambda e_{0}$ is approximately conserved under stable growth (as derived in Subsection 2.3). In real populations, vital rates vary over time and migration occurs. Nevertheless, when $\lambda e_{0}$ is empirically close to constant, growth rates are also near-constant (as shown later in Fig. 3(b)), making the stable-population benchmark a useful first-order approximation. In contrast, if other factors such as educational investment drive fertility decline, $\lambda e_{0}$ need not be conserved, and different combinations of demographic variables may be conserved instead. Identifying such conserved quantities helps to distinguish the primary mechanisms shaping demographic transitions and to assess how broadly they apply.

By analysing global data, this study identifies recurrent regularities in the joint dynamics of fertility and longevity across countries. Most countries’ trajectories in the $\left(\lambda ,e_{0}\right)$ plane are well described by one of two common curves, each corresponding to an approximately conserved quantity (e.g., $\lambda e_{0}≃const.$). Following usage in statistical physics, I refer to these curves as ‘master curves’, emphasising that diverse country-specific trajectories can be summarised by a small number of system-independent patterns, suggestive of shared underlying mechanisms. Because national trajectories tend to move along these curves, I also call them ‘universal pathways’, repeatable macroscopic routes in the $\left(\lambda ,e_{0}\right)$ plane. Thus, this study demonstrates that demographic transition comprises two distinct phases, each characterised by its own pathway and dominant mechanism. The recurrence of these phases suggests that much of the global diversity in demographic transition can be explained by two fundamental mechanisms, despite large differences in political systems and historical contexts.

In this study, I ask whether simple cross-national regularities govern the joint change of the crude birth rate λ and period life expectancy at birth $e_{0}$. I first describe the data and analytical methods, including the identification of universal pathways and their conserved quantities. I then characterise the social and economic profiles associated with each pathway and interpret them using a simple parental-investment model with a trade-off between fertility and education. Finally, I relate these results to existing demographic theories, providing an integrative framework that places explanations (i)–(iii) in a common quantitative perspective.

## 2. Methods

### 2.1. Data

The datasets analysed in this study were obtained from the Human Mortality Database (HMD; covering demographic data of developed countries since the 19th century) (Max Planck Institute for Demographic Research et al., 2024) and the United Nations Statistics Division (UNSD; covering most countries and territories since 1950) (United Nations Statistics Division, 2024). The combined dataset covers 237 countries and territories from 1800 to 2020, although coverage varies by source and country, and some data points are missing.

**Figure 1.**
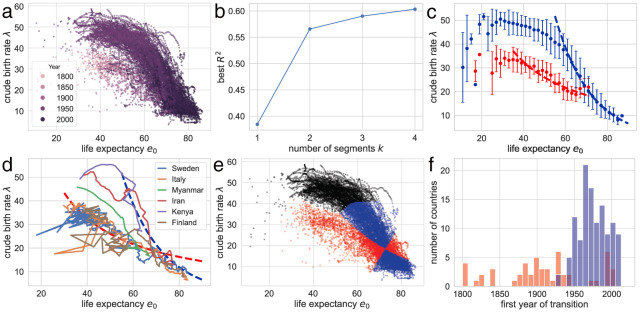
Relationship between the crude birth rate λ and life expectancy at birth $e_{0}$. (a) Scatterplot of data from 237 countries and territories (1800–2020), with colours indicating years. (b) $R^{2}$ as a function of the number of segments k. For each k, the data points are partitioned into k calendar-year intervals, each interval is fit independently, and the maximal $R^{2}$ over breakpoint sets (searched on a 1-year grid) is reported. (c) Two observed trends: pre-1950 (red) and post-1950 (blue). Points represent the average λ within two-year bins of $e_{0}$, with error bars indicating standard deviations. Dashed lines show the isoclines of $\lambda e_{0}$ (red) and $\lambda \exp(e_{0}/17)$ (blue). (d) Sample pathways of countries, with dashed lines as isoclines from (c). The complete list is available in Figs. S3–S10. (e) Data classification into three categories: Phase I (red), closer to the red dashed line; Phase II (blue), closer to the blue dashed line; and pre-transition (black), closer to λ=43. (f) Onset years of each phase. Red and blue histograms indicate the number of countries entering Phase I and Phase II, respectively. Figure 1 long description.

In this study, the crude birth rate is used as a measure of fertility rather than the total fertility rate (TFR), which is commonly employed in demographic research (Bongaarts, 2009; Lesthaeghe, 2014). The TFR is calculated as the sum of age-specific fertility rates for women aged 15–49, and represents the hypothetical number of children a woman would bear if she were exposed throughout her reproductive ages to the age-specific fertility rates observed in a given year. Notably, a TFR of approximately 2 is only a rough benchmark for replacement-level fertility, because exact replacement also depends on survival through the reproductive ages and the timing of childbearing. In contrast, the crude birth rate, which counts the number of births, provides a direct measure of population dynamics. Nonetheless, the comparison of Fig. 1(c) and Fig. S1(b) demonstrates that the qualitative trends are similar when using TFR.

In this study, I primarily rely on the HMD and UNSD datasets because of their transparent metadata and high data quality. For comparison, I repeated key analyses using the Gapminder dataset (https://www.gapminder.org/data/), which offers broader temporal and cross-national coverage but less transparent metadata and quality assurance; the qualitative two-phase structure is unchanged (Fig. S1). Socioeconomic indicators in Fig. 3 are taken from Gapminder, as these variables are outside the scope of HMD/UNSD. Still, Gapminder is used here only for descriptive phase characterisation.

### 2.2. Segmented regression analysis to identify phases of demographic transition

To identify the dominant macroscopic trends governing demographic transition, I apply segmented regression to the relationship between the crude birth rate λ and life expectancy at birth $e_{0}$. Ignoring country labels and segmenting only by calendar year, I search simultaneously over the number of segments k, their breakpoint years, and the curve parameters per segment, testing whether a small number of global curves can describe the entire $\left(\lambda ,e_{0}\right)$ observations. The procedure identifies a two-segment solution with a single change point near 1950, whose robustness I verify by *ex-post* comparison with country-specific trajectories (Figs. S3–S10), a complementary breakpoint-free fit of three pathways, a permutation null, and out-of-sample cross-validation across countries.

For a candidate number of segments k∈{1,…,4}, I search over breakpoint years $\left(t_{1},\ldots ,t_{k-1}\right)$ on a 1-year grid, with $t_{0}=1800$ and $t_{k}=2020$, defining k periods $\left(t_{i-1}\text{--}t_{i}\right)$. For each period, I fit two candidate forms, a power law $\lambda =C_{i}/e_{0}^{\alpha_{i}}$ and an exponential $\lambda =C_{i}/\exp(\beta_{i}e_{0})$, by a grid search over $\alpha_{i}$ (or $\beta_{i}$), with $C_{i}$ estimated by least squares at each candidate. I then retain, for each period, the form and parameter values $\left(\alpha_{i},C_{i}\right)$ (or $\left(\beta_{i},C_{i}\right)$) that minimise the segment-wise sum of squared errors (SSE).

Summing the segment-wise SSEs yields ${SSE}_{total}$, and I evaluate the overall fit by

$$R^{2}=1-{SSE}_{total}/{TSS}_{total},$$

where ${TSS}_{total}$ is the total sum of squared deviations of λ from its overall mean. For each k, I select the breakpoint set $\left(t_{1},\ldots ,t_{k-1}\right)$ that maximises $R^{2}$. I then compare the maximal $R^{2}$ across k and choose the smallest k for which further segmentation yields only marginal improvement (Fig. 1(b)). This procedure supports a two-segment solution with a single change point around $t_{1}≃1950$. Under this specification, the pre-1950 decline in fertility, termed Phase I, is well described by $\lambda e_{0}=C_{1}$, whereas the post-1950 decline, termed Phase II, closely follows $\lambda \exp(e_{0}/17)=C_{2}$. In addition, observations before the onset of fertility decline cluster around a high-fertility plateau $\lambda =C_{0}$.

As a complementary analysis that does not rely on any breakpoint year, I evaluate how well all observations in the $\left(\lambda ,e_{0}\right)$ plane can be explained by the three pathways above: the high-fertility plateau, Phase I, and Phase II. In this analysis, I ignore both country and year labels. I fix the two declining curves to the segmented-regression estimates and determine the plateau level $C_{0}$ by grid search. For each candidate $C_{0}$, each observation is assigned to its closest pathway, and the overall $R^{2}$ is computed. I then retain the value of $C_{0}$ that maximises $R^{2}$ and use this maximised $R^{2}$ to quantify the explanatory power of the three-pathway representation.

To test whether the observed alignment could arise by chance, I construct a permutation null by randomly shuffling λ across observations while keeping $e_{0}$ fixed. For each permuted dataset, I recompute $R^{2}$ after re-optimising the plateau level $C_{0}$ and the two declining pathways, $\lambda e_{0}^{\alpha_{1}}=C_{1}$ and $\lambda \exp(e_{0}/\beta_{2})=C_{2}$. This procedure provides a null distribution for the best achievable three-pathway fit when the empirical association between fertility and longevity is removed.

Finally, to evaluate out-of-sample generalisation across countries, I perform group-wise cross-validation. Countries are split into folds; the pathway parameters are estimated on the training countries by segmented regression; and $R^{2}$ is computed on held-out countries. This cross-validation tests whether pathway parameters learned from one set of countries generalise to previously unseen countries, rather than merely fitting pooled data post hoc.

### Analytic note: why $\lambda e_{0}$ is almost conserved under stable population growth

2.3.

This subsection provides an analytic note on the macroscopic λ–$e_{0}$ pattern under deliberately idealised conditions. I assume a stable population in the standard demographic sense: age-specific fertility and mortality rates are constant over time, and net migration is zero at all ages. Under these assumptions, the population grows exponentially at a constant rate r (Preston et al., 2000, p. 141). This calculation is not intended to predict growth during demographic transition, when vital rates, migration, and age structure vary over time. Rather, it provides a first-order reference relationship between λ and $e_{0}$, explaining why growth is naturally linked to $\lambda e_{0}$ rather than to alternative scalings such as $\lambda e_{0}^{2}$ or $\lambda \exp(e_{0})$. The empirical identification of the two phases remains descriptive and does not rely on the stable-population assumptions.

In a stable population with a small growth rate r (per year), the crude birth rate λ (births per 1,000 individuals per year) and the period life expectancy at birth $e_{0}$ satisfy the following approximation to first order in r (Schindler et al., 2012):

(1) $$\frac{\lambda }{1000}=\frac{1}{e_{0}}+\frac{r}{2}\left(1+\frac{\sigma_{D}^{2}}{e_{0}^{2}}\right),$$

where $\sigma_{D}^{2}$ denotes the variance of age at death. Here, λ/1000 represents the number of births per person per year. Empirically, the ratio $\sigma_{D}^{2}/e_{0}^{2}$ is typically smaller than 1, implying $1<1+\sigma_{D}^{2}/e_{0}^{2}<2$ (Schindler et al., 2012). It then follows that

(2) $$\frac{r}{2}<\frac{\lambda e_{0}/1000-1}{e_{0}}<r.$$

Hence, $(\lambda e_{0}/1000-1)/e_{0}$ provides a rough measure for the growth rate r in this idealised setting. When the population growth rate is small, $\lambda e_{0}/1000$ deviates from 1 by approximately $re_{0}$. Thus, under stable population growth with small r, $\lambda e_{0}$ remains close to 1000 and approximately constant.

### 2.4. Parental investment model with trade-off between fertility and education

To explain the origin of the two pathways, I introduce a simple model of parental investment in children. In the empirical analysis, λ denotes the crude birth rate, measured as births per 1,000 person-years. In the model, I denote relative household fertility by b, which is proportional to λ. Life expectancy $e_{0}$ is taken as given, because mortality decline is often regarded as an exogenous driver of fertility decline (Canning, 2011; Dyson, 2010). The household chooses b and p, where p denotes the fraction of the child’s lifetime allocated to education.

The model represents an economic optimisation problem: households are assumed to maximise the total productive output of their children, not genetic fitness or the number of offspring. This assumption is motivated by evidence that very low fertility in post-industrial populations is not well predicted by fitness maximisation alone, and that lower fertility can increase descendants’ socioeconomic position while reducing long-run genetic fitness (Goodman et al., 2012; Sear et al., 2016). Educational investment is therefore modelled as increasing economic productivity. Under a purely biological objective of maximising offspring number in the present model, the optimum would be p=0. Within this no-education regime, fertility variation follows the classical fertility–longevity trade-off in evolutionary life-history theory, including the r/K continuum and lifetime reproductive effort invariants (Charnov et al., 2007; Stearns, 1992). By contrast, when p>0, fertility declines below this continuum because resources are reallocated from reproduction to education. The present model reproduces a no-education regime when p=0, while also allowing a transition to a positive-education regime when p>0.

Children devote a duration $pe_{0}$ to education, while the remaining $(1-p)e_{0}$ is allocated to productive activities. A unit cost is incurred annually for a child’s survival. Hence, if b children live for $e_{0}$ years, the living cost is $be_{0}$. Parents can also invest in children’s education to improve their productivity. I assume that both the cost and return of education increase approximately exponentially with the duration of education, following the empirical patterns in Fig. 3(e, f). Such a nonlinear increase is reasonable, considering that higher education often requires more specialised personnel and materials.

The educational cost is modelled as $c\exp(\beta pe_{0})-c$, where β determines the degree of nonlinearity and c represents the unit educational cost. Similarly, production efficiency increases by $\alpha \exp(\beta pe_{0})-\alpha$, where α is the unit increment in production efficiency due to education. The terms c and α are subtracted so that both the cost and the productivity increment are zero when p=0.

The household chooses b and p to maximise the total productivity of children subject to the cost constraint

(3) $$b(e_{0}+c\exp(\beta pe_{0})-c)\leq C.$$

Since the optimal b scales linearly with C, I set C=1 without loss of generality. Then, b should be interpreted as relative household fertility rather than an absolute number of children. Under this constraint, b and p are chosen to maximise

(4) $$\max_{b,\;p}b(1-p)e_{0}(1+\alpha \exp(\beta pe_{0})-\alpha ).$$

Because the objective is proportional to b, the constraint is binding at the optimum, giving

(5) $$b=\frac{1}{e_{0}+c\exp(\beta pe_{0})-c}.$$

The optimisation problem therefore reduces to

(6) $$\max_{p}\;f(p)=\frac{\left(1-p)e_{0}(1+\alpha \exp(\beta pe_{0})-\alpha \right)}{e_{0}+c\exp(\beta pe_{0})-c}.$$

While the values of α, β, and c may differ across policies, institutions, and industrial structures, they are typically beyond the control of individual households. Therefore, I first analyse the dependence of b and p on $e_{0}$ by holding these parameters constant, and then examine the effects of policy interventions by varying α, β, and c. The parameters used in the model are summarised in Table 1.

**Table 1.** Model parameters Table 1 long description.

Symbol	Explanation
$e_{0}$	Life expectancy at birth
α	Increment in production efficiency due to education (educational effect)
β	Degree of nonlinearity in educational investment
c	Unit cost of education
b	Relative household fertility, proportional to the empirical crude birth rate λ
p	Fraction of lifetime allocated to education

In this model, $e_{0}$, α, β, and c are predetermined, while b and p are optimised.

The baseline model assumes that parents bear the living costs of their children throughout life. In reality, individuals typically become economically independent after a certain age and pay their own living costs thereafter. To test whether the results depend on this cost-accounting assumption, I analysed a variant in which parents pay each child’s living costs only up to age 20, while adults bear their own subsistence costs after age 20. In this variant, each household incurs its own adult subsistence cost, $\left(e_{0}-20\right)$, in addition to the survival cost of each child up to age 20 and the cost of that child’s education, giving

(7) $$(e_{0}-20)+b(20+c\exp(\beta pe_{0})-c)\leq C.$$

I also examined variants with fixed pre-productive and post-productive periods, such as a case in which children cannot engage in productive activities before age 10. These assumptions shift the critical parameter values at which the transition between Phase I and Phase II occurs, but leave the qualitative two-phase structure unchanged, as shown in Fig. S13. For simplicity and comparability, I present the baseline model in the main text and report these variants as robustness checks.

## 3. Results

### 3.1. Two universal pathways in demographic transition

Figure 1(a) shows the global demographic trend where crude birth rate λ decreases as life expectancy at birth $e_{0}$ increases, with most countries transitioning from the upper left (low $e_{0}$, high λ) to the lower right (high $e_{0}$, low λ). A closer examination reveals that the data points are primarily concentrated along the edges of a triangle, with vertices approximately at $\left(e_{0},\lambda )=(30,50\right)$, (60,50), and (80,10). Older data (lighter colours) tend to cluster along the edge connecting (30,50) and (80,10), whereas more recent data (darker colours) are distributed along the edge between (60,50) and (80,10).

These findings suggest that the relationship between λ and $e_{0}$ varies across distinct timespans. Segmented regression analysis (described in Subsection 2.2) shows that while dividing the dataset into two segments significantly improves the fit compared to a single-segment model, further segmentation (k=3 or k=4) provides only marginal improvements. This suggests the existence of two primary phases in demographic transition with a single change-point around t=1950.

By separating the data into pre- and post-1950 segments, two distinct trends emerge. Figure 1(c) shows that pre-1950 data cluster around the curve $\lambda e_{0}=1300$ (red dashed line), while post-1950 data align with $\lambda \exp(e_{0}/17)=1316$ (blue dashed line). The former relationship implies an annual population growth rate of $r≃(\lambda e_{0}/1000-1)/e_{0}≃0.01$ (see Subsection 2.3; more precisely, $0.3/e_{0}<r<0.6/e_{0}$ for $30\leq e_{0}\leq 60$). By contrast, $\lambda \exp(e_{0}/17)=1316$ corresponds to a steeper decline in λ with increasing $e_{0}$. The divergence in conserved quantities highlights distinct mechanisms driving these transitions. Based on these findings, two phases of demographic transition are identified: Phase I, which conserves $\lambda e_{0}$, and Phase II, which conserves $\lambda \exp(e_{0}/17)$. The pre-transition observations are concentrated near the high-fertility plateau at λ=43.

The presence of these two trends is robust to alternative datasets, fertility measures, and threshold years. The same qualitative pattern is observed (i) in the Gapminder data, (ii) when using the TFR instead of λ, and (iii) when rescaling λ by the working-age (15–60) population share (Fig. S1). Hence, (iii) suggests that these trends cannot be attributed simply to changes in age structure (e.g., population ageing). Moreover, Fig. S2 shows that the two-phase structure is preserved when t is set to 1930. When t=1910, however, fertility decline before t appears less pronounced, whereas when t=1970, the λ–$e_{0}$ relationships largely overlap before and after t.

As illustrated in Fig. 1(d), many national trajectories move along one of the two curves above; I therefore regard these curves as master curves and refer to them as ‘universal pathways’ of demographic transition. Sweden (blue) and Italy (orange) experienced Phase I before transitioning to Phase II. Both initially followed the red curve downward, and upon reaching the intersection with the blue curve (approximately at λ=20 and $e_{0}=70$), they transitioned along the blue curve, exhibiting a steeper decline. Notably, Sweden has been recognised as a country with recent fertility recoveries (Zaidi & Morgan, 2017). However, Fig. 1(d) suggests that such improvements occur only along the blue master curve. In contrast, Myanmar’s trajectory (green) closely follows the red curve, mirroring the pathway of Western countries with a delay of approximately a century. The trajectories of Iran (red) and Kenya (purple) lie close to the blue curve from the onset of fertility decline, indicating that these countries did not experience Phase I before Phase II. Interestingly, Finland (brown) first underwent Phase I, then saw a sudden rise in both fertility and longevity, and transitioned into Phase II. These examples illustrate common trajectory types. The complete set of country trajectories in Figs. S3–S10 shows that these patterns are not restricted to selected cases.

Based on the distance to the two master curves and the high-fertility plateau, data points are classified into three phases: Phase I (red), Phase II (blue), or the pre-transition phase (black) in Fig. 1(e). Figure 1(f) illustrates that Phase I can occur at any time, indicating its universal nature, whereas Phase II is specific to the modern era. Around 1950, many countries left the Phase I pathway and moved towards Phase II, a transition during which both $e_{0}$ and λ rise transiently. This provides a geometric interpretation of the post-war baby boom: fertility rose after departure from Phase I, but the boom ended once trajectories reached the Phase II pathway, after which λ declined along the blue curve. Tables 2 and S1 present the years in which each country experienced each phase.

**Table 2.** The year in which each country experienced each phase of demographic transition (excerpt) Table 2 long description.

Phase I	Country	Phase II
1800–1805, 1815–1950	Sweden	1951–2020
1896–1970	Italy	1971–2020
1986–2020	Myanmar	1975–1985
1995–2003, 2011–2018	Iran	1986–1994, 2004–2010
—	Kenya	2002–2020
1882–1945, 1951–1969	Finland	1946–1950, 1970–2020

*Notes*: Yearly data up to 2020 were used for the analysis. The complete list is available in Table S1.

Three complementary analyses further show that data points concentrate around the proposed pathways, that this concentration is unlikely under a permutation null, and that the fitted pathways generalise across countries. When all observations are analysed directly in the $\left(\lambda ,e_{0}\right)$ plane while ignoring country and year labels, a three-curve representation (high-fertility plateau, Phase I pathway, and Phase II pathway) explains $R^{2}=0.888$ of the variance in λ across the pooled data. This value is higher than the $R^{2}$ obtained from the original segmented regression in Fig. 1(b), which imposes a single global change point near 1950. The label-free model better captures countries that enter Phase I well after 1950, such as Myanmar. The observed alignment is also unlikely under a null model: a permutation test that shuffles λ across observations while keeping $e_{0}$ fixed yields substantially smaller $R^{2}$, and the observed value is exceeded in only p<0.0001 of permutations. Finally, the fit generalises across countries in group-wise cross-validation (mean out-of-sample $R^{2}=0.886$). Together, these results quantify dispersion around the proposed pathways directly and support the hypothesis that demographic transition is organised by two universal pathways in the $\left(\lambda ,e_{0}\right)$ plane.

**Figure 2.**
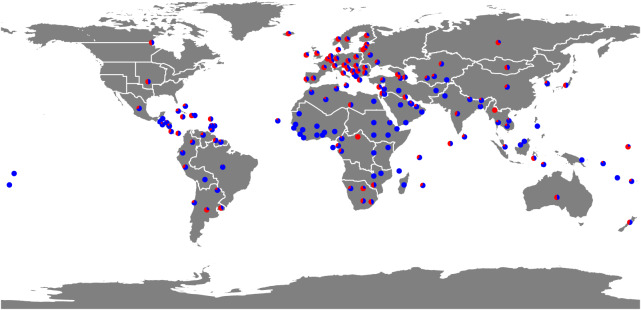
The distribution of countries experiencing Phases I and II. The pie charts illustrate the proportion of years in Phase I (red) and Phase II (blue). Figure 2 long description.

Figure 2 illustrates the geographical distribution of phases. Phase I is prevalent in, though not exclusive to, Western Europe, North America, and East Asia, with most transitioning to Phase II after 1950. In contrast, the majority of countries in Africa, South America, and South Asia have experienced only Phase II. Phase I is typically observed in countries where fertility declined until the mid-20th century. However, some countries, such as Myanmar (Fig. 1(d)), experienced it in the latter half of the 20th century.

### 3.2. Two phases of demographic transition

**Figure 3.**
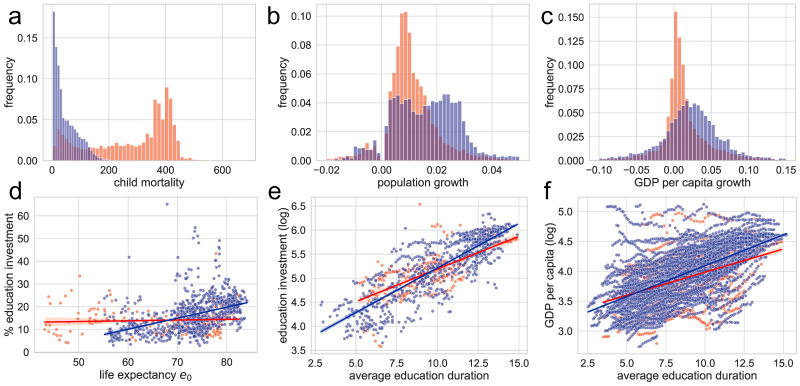
Indices characterising the two phases of demographic transition. The histograms present (a) child mortality rate (average number of deaths of children under five per 1,000 births), (b) population growth rate, and (c) GDP per capita growth rate. Scatterplots (d)–(f) illustrate relationships between demographic and educational indicators: (d) life expectancy at birth $e_{0}$ versus education investment per student relative to GDP per capita, (e) average educational duration versus education investment per student (log scale, USD), and (f) average educational duration versus GDP per capita, representing the return on educational investment. Red and blue indicate data corresponding to Phases I and II, respectively. The red and blue lines in (d)–(f) represent linear regression results. Indicators in this figure are taken from the Gapminder dataset; phase labels are based on the HMD/UNSD analysis. Figure 3 long description.

Figure 3 shows the distinct features of the two phases. Phase I is characterised by high child mortality and a steady but low rate of population growth, as shown in Fig. 3(a, b). This indicates that population growth rates are close to 1% for most countries and years, supporting the interpretation that the approximate conservation of $\lambda e_{0}$ during Phase I reflects nearly constant population growth. In contrast, Fig. 3(a, c) shows that Phase II is marked by lower child mortality and steady growth in GDP per capita. Population growth continues in Phase II, but its rate varies widely across countries and years (Fig. 3(b)), while GDP per capita grows steadily at around 2%. Thus, the macroscopic regularity of Phase II is more closely associated with steady per-capita GDP growth than with steady population growth. Moreover, Fig. S11 shows that although $e_{0}$ increases at a similar pace in both phases, the decline of λ is larger in Phase II.

Educational investment is more prominent in Phase II than in Phase I. Figure 3(d) plots per-student education expenditure divided by GDP per capita against $e_{0}$. This share remains low and nearly independent of $e_{0}$ in Phase I (corr. =0.08), whereas in Phase II it rises with $e_{0}$ (corr. =0.43). GDP per capita itself grows approximately exponentially with $e_{0}$ in both phases, but with a steeper exponent in Phase II (Fig. S12(b)). Consequently, absolute educational investment per student also increases approximately exponentially with $e_{0}$; the correlation between $e_{0}$ and log-transformed investment is stronger in Phase II (corr. =0.86) than in Phase I (corr. =0.56), as shown in Fig. S12(c). As many countries shift from Phase I to Phase II around $e_{0}\approx 70$, near the intersection of the two pathways, this transition is accompanied by a sharp acceleration in educational investment. Additionally, Fig. 3(e, f) indicates that, in both phases, educational investment and GDP per capita, representing the return on investment, increase approximately exponentially with education duration.

These results also clarify how the two pathways relate to the conventional distinction between the first and second demographic transitions. The educational and economic signatures of Phase II align with the broader societal shift towards ‘higher-order needs’, such as self-realisation, expressive work and educational values, which characterise the second demographic transition (Inglehart, 2018; Lesthaeghe, 2014; Maslow, 1954). They also reflect the ‘quantity–quality trade-off’ in economics and human behavioural ecology, whereby fertility is balanced against investment in human capital through education (Colleran et al., 2015; Fernihough, 2017; Hanushek, 1992; Kaplan, 1996).

Phase I, the movement along the red curve in Fig. 1(c), is broadly consistent with the first demographic transition, which is typically characterised by a decline in fertility towards the replacement level. This connection can be seen by relating the present variables to the TFR, a standard measure of fertility in demography. The TFR is a period measure, defined as the number of children a woman would bear on average if she were exposed throughout her reproductive ages (15–49) to the age-specific fertility rates observed in a given calendar year. Realised cohort fertility can differ from the TFR because of mortality before or during the reproductive ages and tempo effects such as delayed childbearing. Nevertheless, along the Phase I pathway, rising $e_{0}$ reduces mortality before and during the reproductive ages, and the steady low-growth condition brings populations towards replacement-level fertility. Consistently, the corresponding trajectory in the TFR–$e_{0}$ plane approaches TFR≃2 (Fig. S1).

By contrast, the observed trends in Phase II highlight the limitations of categorising demographic transitions solely based on TFR. In Fig. 1(c), the segment of the blue curve of Phase II below the red curve of Phase I signifies a fertility decline below the replacement level, defining the second demographic transition. However, observations in Phase II that lie above the red curve, which are often found in developing countries, present a puzzle. Traditional classifications attribute these states to the first demographic transition, as TFR exceeds 2. Yet, the present findings suggest that these states align more closely with the second demographic transition in Western countries, as they follow the same universal pathway.

These two phases are also consistent with the growth phases proposed in the unified growth theory (Galor, 2011). Phase I corresponds to the Malthusian growth phase, characterised by steady population growth, while Phase II corresponds to the modern growth phase, which is marked by sustained growth in GDP per capita and technological levels.

### 3.3. Origin of universal pathways

I next examine the origin of the two universal pathways. As described in Subsection 2.3, $\lambda e_{0}$ remains conserved under conditions of stable population growth. Thus, Phase I follows the Malthusian growth model, where fertility decline is driven by population pressure. This scenario can be interpreted as the optimisation of the total quantity of people. In contrast, when the focus shifts to optimising their total ‘quality’, a different phase will emerge.

A simple model of parental investment in children is considered to elucidate the transition between Phases I and II. In this model, life expectancy $e_{0}$ is assumed to be given. Children devote a duration of $pe_{0}$ to education, while the remaining $(1-p)e_{0}$ is allocated to productive activities. Children’s living cost is given by $be_{0}$. Here, b denotes relative household fertility, which is proportional to λ. The educational cost is modelled as $c\exp(\beta pe_{0})-c$, where β determines the degree of nonlinearity, and c represents the unit educational cost. Similarly, production efficiency increases by $\alpha \exp(\beta pe_{0})-\alpha$, where α is the unit increment in production efficiency due to education. Then, b and the fraction of the child’s lifetime allocated to education p are optimised to maximise the total productivity of children. (See Subsection 2.4 for details.) As b is proportional to λ, any conserved quantity involving b in the model corresponds to an analogous conserved quantity involving λ, up to a constant multiplicative factor.

**Figure 4.**
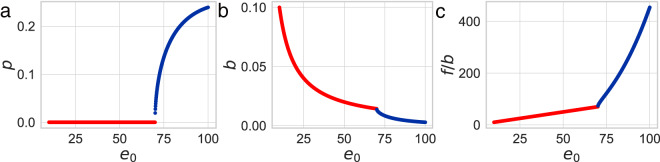
Results of numerical calculations. (a) The optimal fraction of educational duration p, (b) the optimal fertility b, and (c) per-child productivity f/b as functions of life expectancy $e_{0}$. In this calculation, the parameters are set to α=0.5, β=0.1, and c=25. Points for Phase I (p=0) are plotted in red, while those for Phase II (p>0) are plotted in blue.

Numerical calculations in Fig. 4 reveal that as life expectancy $e_{0}$ increases, educational investment for children becomes suddenly advantageous, delineating two distinct phases. Figure 4(a) shows a sudden rise in the optimal p at a specific $e_{0}$ threshold. For small $e_{0}$, the optimal p equals zero, conserving $be_{0}$. Because b∝λ, this model regularity corresponds to the empirical Phase I regularity $\lambda e_{0}≃const.$ In this phase, parents allocate resources to maximise the number of children without educational investment.

As $e_{0}$ increases, longer lifespans enable extended productive work, making educational investment more feasible. Consequently, the optimal p becomes positive and remains relatively stable, leading to a gradual increase in the optimal educational duration $pe_{0}$. Then, by the cost constraint, $b\left(c\exp(\beta pe_{0})+e_{0}-c\right)≃bc\exp(\beta pe_{0})$ is conserved. Thus, once educational investment is adopted, fertility declines approximately exponentially with life expectancy. This model regularity corresponds to the empirical Phase II regularity $\lambda \exp(e_{0}/17)≃const.$, with 1/17 interpreted as an effective value of βp. The phase shift aligns with Fig. 3(d), where educational investment transitions from being minimal and $e_{0}$-independent in Phase I to increasing exponentially with $e_{0}$ in Phase II.

**Figure 5.**
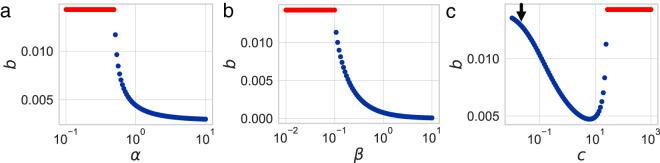
Dependence of the optimal fertility b on (a) the educational effect α, (b) the nonlinearity in educational investment β, and (c) the educational cost c. Unless otherwise specified, the parameters are set to α=0.5, β=0.1, c=25, and $e_{0}=70$. Points for Phase I (p=0) are plotted in red, while those for Phase II (p>0) are plotted in blue. The downward arrow in (c) represents the proposed policy intervention to enhance fertility.

Note that the exact value of the optimal p and the $e_{0}$ threshold at which the transition occurs depend on the specific parameter values of α, β, and c (as later illustrated in Fig. 5). However, the qualitative phenomenon itself, the abrupt increase of p from zero to a positive value at a certain $e_{0}$, is robust and emerges regardless of the particular parameter settings. This qualitative behaviour also persists in model variants where adults pay their own living costs and where pre-productive and post-productive periods are considered (Fig. S13).

The optimal fertility in Fig. 4(b) illustrates a seamless and spontaneous transition from Phase I to Phase II as a function of $e_{0}$. This resembles the trajectories of many Western countries (e.g., Italy and Sweden in Fig. 1(d)). By contrast, extrapolating the right-hand branch where p>0 reproduces the blue master curve in Fig. 1(c). The trajectories of many developing countries, which either did not experience Phase I or exited it midway, follow this extrapolated blue curve. This suggests that their demographic dynamics were influenced by cultural transmission or Westernisation (Amin et al., 2002; Colleran, 2016; Lesthaeghe, 2010). In other words, their demographic transition may have resulted from the adoption of institutional parameters (α,β, and c) and/or behavioural strategies prioritising child education over fertility, imported from Western countries in Phase II.

Finally, Fig. 4(c) illustrates per-child productivity f/b, used as a proxy for GDP per capita. It remains nearly constant within Phase I and increases steadily with $e_{0}$ during Phase II, consistent with the trends observed in Fig. 3(c).

Next, the impact of various policies on optimal fertility is examined by analysing its dependence on parameters. By fixing $e_{0}$ at 70 and varying the values of α, β, and c, the optimal value of b is determined. Figure 5 shows that fertility is highest when the educational effect α and the nonlinearity in educational investment β are small, while the educational cost c is high. However, this region corresponds to Phase I, where p=0. This scenario, characterised by ineffective educational investment, represents an undesirable outcome from a policy perspective.

Conversely, Fig. 5(c) indicates that a significant reduction in the educational cost, c, can increase fertility (as indicated by the arrow), while remaining in Phase II. This suggests that making education more affordable allows resources to be reallocated towards reproduction without lowering educational standards. Such a scenario is favourable, as it enables both the maintenance of a healthy population growth rate and sustained investment in education. Moreover, global data in Fig. S14 support this observation, showing that lower values of c are associated with higher fertility rates.

## 4. Discussion

### 4.1. Universal mechanisms of demographic transition

By examining the relationship between the crude birth rate λ and life expectancy at birth $e_{0}$, two universal pathways of demographic transition have been identified, characterised by the conservation of $\lambda e_{0}$ and $\lambda \exp(e_{0}/17)$, respectively. These pathways define two distinct phases: Phase I, corresponding to the first demographic transition in Western countries and the Malthusian growth phase; and Phase II, corresponding to the first demographic transition in most developing countries and to the second demographic transition across regions, consistent with the modern growth phase (Galor, 2011; Lesthaeghe, 2014; Zaidi & Morgan, 2017). Although some studies associate the modern growth phase with the first demographic transition (Zaidi & Morgan, 2017), the present findings suggest that it more accurately coincides with the second. The current contribution is to make the two-phase structure explicit as two qualitatively distinct macroscopic regularities in the dynamics of fertility and longevity, and to provide a mechanistic interpretation for why these regularities arise.

Importantly, this universality should not be interpreted as a unilinear stage theory. Recent critiques of the second demographic transition have argued that stage-based interpretations can become overly developmental, place too much explanatory weight on ideational change, and insufficiently account for persistent within-country heterogeneity (Zaidi & Morgan, 2017). The present results do not imply that all societies follow a single irreversible sequence towards a common end state. Rather, they show that, at the macroscopic level of the joint dynamics of λ and $e_{0}$, two recurrent pathways account for a large share of the observed cross-national variation. In this sense, the contribution of the present study is to identify a simple quantitative regularity that is compatible with multiple country-specific trajectories and mechanisms, while still suggesting that a limited number of dominant processes may generate shared patterns.

Phase I has occurred at any time over the past 200 years, whereas Phase II is unique to the modern era, emerging predominantly after 1950. Phase I was prevalent in Western Europe, North America, and East Asia, characterised by high child mortality and steady population growth. Most of these regions transitioned to Phase II after 1950. In contrast, most countries in Africa, South America, and South Asia experienced only Phase II, marked by low child mortality and steady growth in GDP per capita. This raises questions about the applicability of the first and second demographic transition frameworks outside of Western contexts.

Interestingly, vertebrates also exhibit a linear relationship between fertility and mortality, conserving $\lambda e_{0}$, as in Phase I (Ricklefs, 2010). Within Phase I, populations with high fertility and high mortality correspond to r-selected life histories, whereas those with low fertility and low mortality correspond to K-selected life histories. Phase II, by contrast, represents a state in which fertility has fallen well below this continuum, departing from r/K selection. This contrast suggests that Phase I reflects a universal life-history pattern in nature, whereas Phase II is a uniquely human phenomenon.

A simple model was then proposed by considering the trade-off between reproduction and education. Numerical analysis demonstrated the transition between Phase I, where reproduction is prioritised with minimal investment in education, and Phase II, where education is emphasised even at the cost of reduced fertility. Phase I emerges when life expectancy is relatively low, and education is costly or yields modest productivity gains, leading to a no-education solution (p=0) that conserves $\lambda e_{0}$. As $e_{0}$ rises and education becomes more affordable and effective, the optimum shifts to a positive-education solution (p>0), yielding Phase II with conservation of $\lambda \exp(\beta pe_{0})$. Thus, in Phase II, fertility decreases exponentially with increasing life expectancy. Here, β represents the nonlinearity in educational investment, and p denotes the optimal fraction of educational duration per lifetime. This phase transition corresponds to the shift from the first to the second demographic transition and from the Malthusian to the modern growth phase (Galor, 2011; Lesthaeghe, 2014).

The parameter dependence of optimal fertility provides a common lens for interpreting both historical change and policy intervention. The theoretical results align with sociological observations that modernisation facilitated greater social mobility through lower-cost mass education, supported by a meritocratic ideology (Breen & Jonsson, 2007; Gil-Hernández et al., 2017; Squicciarini & Voigtländer, 2016). In terms of the model, this institutional transformation would have incentivised educational investment by increasing the educational effect α and reducing the educational cost c, thereby promoting the emergence of Phase II. The same parameter dependence also carries policy implications: lowering educational cost c can reallocate resources towards reproduction while preserving educational standards. Hence, policies that reduce the financial burden of education may support fertility recovery without compromising human capital development.

### 4.2. Connection to demographic theories

This study reveals universal macroscopic regularities in demographic transition by identifying conserved quantities during dynamic changes, a method commonly employed in statistical physics. The approximate conservation of $\lambda e_{0}$ and $\lambda \exp(e_{0}/17)$ provides empirical criteria that any satisfactory explanation of the macroscopic structure of demographic transition should satisfy. Therefore, the present findings provide empirical constraints for advancing the theory of demographic transition.

Previous explanations of fertility decline can be broadly classified into three categories based on their primary focus: (i) population pressure (Malthus, 1798), (ii) the trade-off between education and fertility (Becker et al., 2010; Bleakley & Lange, 2009; Canning, 2011; Fernihough, 2017; Galor, 2011; Hanushek, 1992; Kaplan, 1996; Lawson & Mace, 2011; Mace, 2000; Shenk, 2009; Werding, 2014), and (iii) the cultural transmission of behavioural strategies (Amin et al., 2002; Ihara and Feldman, 2004; Mulder, 1998; Murphy, 2017; Newson et al., 2007; Richerson & Boyd, 2008). The present framework integrates these perspectives by identifying (i) as the driver of Phase I, (ii) as that of Phase II, and (iii) as that of the transition from Phase I to Phase II.

Rapid fertility decline in many developing regions has often been linked to Westernisation (Kirk, 1996; Murphy, 2017). The present findings suggest that Westernisation may have facilitated the cultural transmission of education-oriented parental investment norms or the adoption of institutional parameters such as educational effect α, the nonlinearity of educational investment β, and educational cost c, thereby accelerating the transition to Phase II. These processes, also highlighted in previous studies (Amin et al., 2002; Borenstein et al., 2006), help explain why many developing countries entered Phase II directly or exited Phase I midway.

Educational investment as a driver of fertility decline in Phase II offers additional insights. As child mortality declines, a trend evident in Phase II as shown in Fig. 3(a), longevity becomes more predictable (Aburto et al., 2020). This increased predictability has been proposed to promote fertility decline by reducing the perceived risk of lineage extinction (Leslie and Winterhalder, 2002). The present theory further suggests that with more predictable longevity, the incentive to invest in education intensifies, thereby accelerating the transition to Phase II. Moreover, extended educational attainment and subsequent labour force participation, particularly among women, have been associated with delayed first births, accelerating fertility decline (Snopkowski et al., 2016).

The present analysis identifies two quantities that remain approximately constant during demographic transition, but it does not explain why they take their particular numerical values. In Phase I, the conserved quantity is $\lambda e_{0}$: as life expectancy increases, the crude birth rate declines in inverse proportion. The observed value $\lambda e_{0}≃1300$ corresponds approximately to steady population growth at an annual rate of around 1%. Why many countries share this growth rate remains unclear. In Phase II, by contrast, the conserved quantity is $\lambda \exp(e_{0}/17)$, implying an approximately exponential decline in fertility with increasing life expectancy. Yet the values 17 and 1316 in $\lambda \exp(e_{0}/17)≃1316$ remain unexplained. It would be unrealistic to assume that household cost constraints, or effective carrying capacities, increase at nearly the same rate across countries. A more plausible interpretation is that biological and sociocultural factors limit how far additional resources can be converted into higher fertility and net population growth, so that countries differ mainly in how long they remain near this upper bound, rather than in the level of the bound itself. Explaining the numerical values of these conserved quantities remains an open question.

Additionally, several limitations should be acknowledged. First, data coverage is limited. HMD primarily covers developed countries, whereas UNSD data span only the post-war period. In addition, as the socioeconomic measures were limited in these databases, the characterisation in Fig. 3 relies on Gapminder, which offers broad coverage, albeit with variable reliability. The insights from this study should therefore be tested using future datasets with broader and more reliable coverage. Second, the current model focuses on the trade-off between reproduction and education, and is therefore not designed to capture the full range of constraints that may shape fertility behaviour within each phase, including trade-offs involving parents’ career advancement and leisure, as well as labour-market uncertainty (Debnath & Das, 2022; Galindev, 2011; Hakim, 2003; Hellstrand et al., 2024; Kato, 2021; Vitali et al., 2009). During recent fertility decline in Finland, tertiary educational attainment among young adults has stagnated (OECD, 2025). These observations suggest that fertility decline in Phase II need not always be accompanied by continued educational expansion. Future work should therefore clarify whether such heterogeneity reflects additional trade-offs embedded within Phase II or signals the emergence of another phase. More broadly, it remains unclear why some countries, such as Finland, transitioned from Phase I to Phase II midway, while others (e.g., Italy and Sweden) stayed in Phase I until reaching the intersection of the two pathways.

In conclusion, this study identifies two universal pathways in demographic transition by analysing the relationship between the crude birth rate λ and period life expectancy at birth $e_{0}$ across countries worldwide. These pathways, characterised by the conservation of $\lambda e_{0}$ and $\lambda \exp(e_{0}/17)$, correspond to phases where reproduction or education is prioritised. This work provides a novel perspective on demographic transition by uncovering universal patterns from global data and integrating them with a simple theoretical framework.

## Supporting information

10.1017/ehs.2026.10054.sm001 Itao supplementary material 1 Itao supplementary material

10.1017/ehs.2026.10054.sm002 Itao supplementary material 2 Itao supplementary material

## Supplementary material

The supplementary material for this article can be found at https://doi.org/10.1017/ehs.2026.10054.

## Research transparency and reproducibility interest

The primary demographic datasets analysed in this study were obtained from the Human Mortality Database (HMD) (Max Planck Institute for Demographic Research et al., 2024) and the United Nations Statistics Division (UNSD) (United Nations Statistics Division, 2024). Gapminder was used for supplementary analyses and for the socioeconomic characterisation of phases (e.g., Fig. 3 and Supplementary Information; https://www.gapminder.org/data/). The source code is available at: https://github.com/KenjiItao/DemographicTransition.git.

## Table 1 Long description

Table 1 defines the variables and parameters used in the parental-investment model. Life expectancy at birth, e0, is treated as a predetermined demographic input. The parameters α, β and c describe educational settings: α is the increment in production efficiency due to education, β is the degree of nonlinearity in educational investment, and c is the unit cost of education. The variable b represents relative household fertility and is proportional to the empirical crude birth rate λ. The variable p represents the fraction of a child’s lifetime allocated to education. In the model, e0, α, β and c are predetermined, while b and p are optimised.

Navigate back to Table 1.

## Figure 1 Long description

Figure 1 summarises the empirical identification of two universal pathways in demographic transition. Panel (a) plots crude birth rate against life expectancy at birth for 237 countries and territories from 1800 to 2020, with point colour indicating calendar year. The data form a triangular pattern: high fertility is concentrated at lower to intermediate life expectancy, while fertility declines as life expectancy rises. Panel (b) shows that segmented regression improves substantially when moving from one to two segments, with only modest additional improvement for three or four segments, supporting a two-phase description. Panel (c) compares the two fitted trends: pre-1950 observations follow an inverse relationship close to λe0 = constant, whereas post-1950 observations follow a steeper decline close to λ exp(e0/17) = constant. Panel (d) shows representative country trajectories for Sweden, Italy, Myanmar, Iran, Kenya and Finland, illustrating that countries may follow Phase I, Phase II, or shift between them. Panel (e) classifies all observations into pre-transition, Phase I and Phase II according to proximity to the high-fertility plateau and the two fitted pathways. Panel (f) shows the onset years of Phase I and Phase II, indicating that Phase I occurs across a broad historical period, whereas Phase II is concentrated mainly in the modern era after the mid-twentieth century.

Navigate back to Figure 1.

## Figure 2 Long description

Figure 2 maps the geographical distribution of demographic phases. Each country or territory is represented by a pie chart showing the proportion of observed years classified as Phase I and Phase II. Red segments indicate years in Phase I, where λe0 is approximately conserved, and blue segments indicate years in Phase II, where λ exp(e0/17) is approximately conserved. Phase I is common but not exclusive to Western Europe, North America and East Asia, many of which later transition to Phase II. In contrast, many countries in Africa, South America and South Asia are dominated by Phase II observations.

Navigate back to Figure 2.

## Figure 3 Long description

Figure 3 compares demographic, economic and educational characteristics of Phase I and Phase II. Panels (a) to (c) show histograms of child mortality (a), population growth (b) and GDP per capita growth (c). Phase I observations are concentrated at higher child mortality and around a low but relatively steady population growth rate. Phase II observations have lower child mortality and more variable population growth, but GDP per capita growth is concentrated around a positive value. Panels (d) to (f) show scatterplots relating life expectancy and education. Panel (d) plots life expectancy at birth against education investment per student relative to GDP per capita; the relationship is weak in Phase I but positive in Phase II. Panel (e) shows that education investment per student on a logarithmic scale increases with average education duration. Panel (f) shows that GDP per capita on a logarithmic scale also increases with average education duration. Together, the panels indicate that Phase I is more closely associated with stable population growth, whereas Phase II is more closely associated with economic growth and increased educational investment.

Navigate back to Figure 3.

## Table 2 Long description

Table 2 gives example country-level classifications for the two demographic phases using yearly data up to 2020. For each selected country, the table lists the years assigned to Phase I and the years assigned to Phase II. Sweden and Italy show long Phase I periods followed by Phase II. Myanmar is shown as experiencing Phase I in 1986–2020 and Phase II in 1975–1985. Iran and Kenya entered Phase II before experiencing Phase I. Finland shows Phase I in 1882–1945 and 1951–1969, and Phase II in 1946–1950 and 1970–2020. The table illustrates that countries can follow different temporal sequences, including transitions between phases or direct entry into Phase II.

Navigate back to Table 2.

## References

[ref1] Aburto, J. M., Villavicencio, F., Basellini, U., Kjærgaard, S., & Vaupel, J. W. (2020). Dynamics of life expectancy and life span equality. *Proceedings of the National Academy of Sciences*, 117(10), 5250–5259. 10.1073/pnas.1915884117 PMC7071894 32094193

[ref2] Amin, S., Basu, A. M., & Stephenson, R. (2002). Spatial variation in contraceptive use in Bangladesh: Looking beyond the borders. *Demography*, 39(2), 251–267. 12048951 10.1353/dem.2002.0014

[ref3] Becker, S. O., Cinnirella, F., & Woessmann, L. (2010). The trade-off between fertility and education: Evidence from before the demographic transition. *Journal of Economic Growth*, 15, 177–204.

[ref4] Bleakley, H., & Lange, F. (2009). Chronic disease burden and the interaction of education, fertility, and growth. *The Review of Economics and Statistics*, 91(1), 52–65. 24163482 10.1162/rest.91.1.52 PMC3806284

[ref5] Bloom, D., Canning, D., & Sevilla, J. (2003). *The demographic dividend: A new perspective on the economic consequences of population change*. Rand Corporation.

[ref6] Bongaarts, J. (2009). Human population growth and the demographic transition. *Philosophical Transactions of the Royal Society B: Biological Sciences*, 364(1532), 2985–2990. 10.1098/rstb.2009.0137 PMC2781829 19770150

[ref7] Borenstein, E., Kendal, J., & Feldman, M. (2006). Cultural niche construction in a metapopulation. *Theoretical Population Biology*, 70(1), 92–104. 16426653 10.1016/j.tpb.2005.10.003

[ref8] Breen, R., & Jonsson, J. O. (2007). Explaining change in social fluidity: Educational equalization and educational expansion in twentieth-century Sweden. *American Journal of Sociology*, 112(6), 1775–1810.

[ref9] Canning, D. (2011). The causes and consequences of demographic transition. *Population Studies*, 65(3), 353–361. 21973179 10.1080/00324728.2011.611372

[ref10] Charnov, E. L., Warne, R., & Moses, M. (2007). Lifetime reproductive effort. *The American Naturalist*, 170(6), E129–E142. 10.1086/522840 18171160

[ref11] Colleran, H. (2016). The cultural evolution of fertility decline. *Philosophical Transactions of the Royal Society B: Biological Sciences*, 371(1692), 20150152. 10.1098/rstb.2015.0152 PMC4822432 27022079

[ref12] Colleran, H., Jasienska, G., Nenko, I, Galbarczyk, A., & Mace, R. (2015). Fertility decline and the changing dynamics of wealth, status and inequality. *Proceedings of the Royal Society B: Biological Sciences*, 282(1806), 20150287. 10.1098/rspb.2015.0287 PMC4426630 25833859

[ref13] Debnath, A., & Das, S. (2022). Inter-relationship among female labour-force participation, fertility and economic development: Evidences from India. *Economic Affairs*, 67(4), 673–680.

[ref14] Dyson, T. (2010). *Population and development: The demographic transition*. Bloomsbury Publishing.

[ref15] Fernihough, A. (2017). Human capital and the quantity–quality trade-off during the demographic transition. *Journal of Economic Growth*, 22, 35–65.

[ref16] Galindev, R. (2011). Leisure goods, education attainment and fertility choice. *Journal of Economic Growth*, 16, 157–181.

[ref17] Galor, O. (2011). *Unified growth theory*. Princeton University Press.

[ref18] Gil-Hernández, C. J., Marqués-Perales, I., & Fachelli, S. (2017). Intergenerational social mobility in Spain between 1956 and 2011: The role of educational expansion and economic modernisation in a late industrialised country. *Research in Social Stratification and Mobility*, 51, 14–27.

[ref19] Goodman, A., Koupil, I, & Lawson, D. W. (2012). Low fertility increases descendant socioeconomic position but reduces long-term fitness in a modern post-industrial society. *Proceedings of the Royal Society B: Biological Sciences*, 279(1746), 4342–4351. 10.1098/rspb.2012.1415 PMC3479798 22933371

[ref20] Hakim, C. (2003). A new approach to explaining fertility patterns: Preference theory. *Population and Development Review*, 29(3), 349–374.

[ref21] Hanushek, E. A. (1992). The trade-off between child quantity and quality. *Journal of Political Economy*, 100(1), 84–117.

[ref22] Hellstrand, J., Nisén, J., & Myrskylä, M. (2024). Educational field, economic uncertainty, and fertility decline in Finland in 2010–2019. *European Sociological Review*, 40(5), 754–771. 39371593 10.1093/esr/jcae001 PMC11451949

[ref23] Ihara, Y., & Feldman, M. W. (2004). Cultural niche construction and the evolution of small family size. *Theoretical Population Biology*, 65(1), 105–111. 14642348 10.1016/j.tpb.2003.07.003

[ref24] Inglehart, R. (2018). *Culture shift in advanced industrial society*. Princeton University Press.

[ref25] Kaplan, H. (1996). A theory of fertility and parental investment in traditional and modern human societies. *American Journal of Physical Anthropology: The Official Publication of the American Association of Physical Anthropologists*, 101(S23), 91–135.

[ref26] Kato, H. (2021). Low Fertility and Female Labor Supply in Japan—Time Series Analysis Using Bayesian VAR Approach. In Kato H (Ed.), *Macro-Econometric Analysis on Determinants of Fertility Behavior*, (pp. 1–23). Singapore: Springer Singapore.

[ref27] Kirk, D. (1996). Demographic transition theory. *Population Studies*, 50(3), 361–387. 11618374 10.1080/0032472031000149536

[ref28] Lawson, D. W., & Mace, R. (2011). Parental investment and the optimization of human family size. *Philosophical Transactions of the Royal Society B: Biological Sciences*, 366(1563), 333–343. 10.1098/rstb.2010.0297 PMC3013477 21199838

[ref29] Leslie, P., & Winterhalder, B. (2002). Demographic consequences of unpredictability in fertility outcomes. *American Journal of Human Biology*, 14(2), 168–183. 11891932 10.1002/ajhb.10044

[ref30] Lesthaeghe, R. (2010). The unfolding story of the second demographic transition. *Population and Development Review*, 36(2), 211–251. 20734551 10.1111/j.1728-4457.2010.00328.x

[ref31] Lesthaeghe, R. (2014). The second demographic transition: A concise overview of its development. *Proceedings of the National Academy of Sciences*, 111(51), 18112–18115. 10.1073/pnas.1420441111 PMC4280616 25453112

[ref32] Lutz, W., Crespo Cuaresma, J., Kebede, E., Prskawetz, A., Sanderson, W. C., & Striessnig, E. (2019). Education rather than age structure brings demographic dividend. *Proceedings of the National Academy of Sciences*, 116(26), 12798–12803. 10.1073/pnas.1820362116 PMC6600906 31182606

[ref33] Mace, R. (2000). Evolutionary ecology of human life history. *Animal Behaviour*, 59(1), 1–10. 10640361 10.1006/anbe.1999.1287

[ref34] Mace, R. (2008). Reproducing in cities. *Science*, 319(5864), 764–766. 18258904 10.1126/science.1153960

[ref35] Malthus, T. R. (1798). *An essay on the principle of population, as it affects the future improvement of society*. Johnson.

[ref36] Maslow, A. H. (1954). *Motivation and personality*. Harper & Row.

[ref37] Max Planck Institute for Demographic Research, University of California, Berkeley, & French Institute for Demographic Studies. (2024). HMD. Human Mortality Database. Retrieved May 28, 2026, from https://www.mortality.org.

[ref38] Mulder, M. B. (1998). The demographic transition: Are we any closer to an evolutionary explanation? *Trends in Ecology & Evolution*, 13(7), 266–270. 21238295 10.1016/s0169-5347(98)01357-3

[ref39] Murphy, M. (2017). *The economization of life*. Duke University Press.

[ref40] Newson, L., Postmes, T., Lea, S. E., Webley, P., Richerson, P. J., & Mcelreath, R. (2007). Influences on communication about reproduction: The cultural evolution of low fertility. *Evolution and Human Behavior*, 28(3), 199–210.

[ref41] Notestein, F. W. (1945). Population–the long view. In Theodore W. Schultz (Ed.), *Food for the World* (pp. 36–57) University of Chicago Press.

[ref42] OECD. (2025). *OECD economic surveys: Finland 2025*. OECD Publishing.

[ref43] Preston, S., Heuveline, P., & Guillot, M. (2000). *Demography: Measuring and modeling population processes*. Wiley.

[ref44] Reher, D. (2012). Population and the economy during the demographic transition. *Economic Affairs*, 32(1), 10–16.

[ref45] Richerson, P. J., & Boyd, R. (2008). *Not by genes alone: How culture transformed human evolution*. University of Chicago Press.

[ref46] Ricklefs, R. E. (2010). Life-history connections to rates of aging in terrestrial vertebrates. *Proceedings of the National Academy of Sciences*, 107(22), 10314–10319. 10.1073/pnas.1005862107 PMC2890449 20479246

[ref47] Schindler, S., Tuljapurkar, S., Gaillard, J.-M., & Coulson, T. (2012). Linking the population growth rate and the age-at-death distribution. *Theoretical Population Biology*, 82(4), 244–252. 23103877 10.1016/j.tpb.2012.09.003 PMC3793508

[ref48] Sear, R. (2015). Evolutionary contributions to the study of human fertility. *Population Studies*, 69(sup1), S39–S55. 25912916 10.1080/00324728.2014.982905

[ref49] Sear, R., Lawson, D. W., Kaplan, H., & Shenk, M. K. (2016). Understanding variation in human fertility: What can we learn from evolutionary demography? *Philosophical Transactions of the Royal Society B: Biological Sciences*, 371(1692), 20150144. 10.1098/rstb.2015.0144 PMC4822424 27022071

[ref50] Shenk, M. K. (2009). Testing three evolutionary models of the demographic transition: Patterns of fertility and age at marriage in urban South India. *American Journal of Human Biology: The Official Journal of the Human Biology Association*, 21(4), 501–511. 10.1002/ajhb.20943 19408251

[ref51] Snopkowski, K., Towner, M. C., Shenk, M. K., & Colleran, H. (2016). Pathways from education to fertility decline: A multi-site comparative study. *Philosophical Transactions of the Royal Society B: Biological Sciences*, 371(1692), 20150156. 10.1098/rstb.2015.0156 PMC4822436 27022083

[ref52] Squicciarini, M. P., & Voigtländer, N. (2016). Knowledge elites and modernization: Evidence from Revolutionary France. Technical report. National Bureau of Economic Research.

[ref53] Stearns, S. C. (1992). *The evolution of life histories*. Oxford University Press.

[ref54] United Nations Statistics Division. (2024). Population and vital statistics report, vol. LXXVI. Retrieved May 28, 2026, from https://unstats.un.org/unsd/demographic-social/products/vitstats/.

[ref55] Vitali, A., Billari, F. C., Prskawetz, A., & Testa, M. R. (2009). Preference theory and low fertility: A comparative perspective. *European Journal of Population/Revue européEnne de démographie*, 25, 413–438.

[ref56] Werding, M. (2014). Children are costly, but raising them may pay: The economic approach to fertility. *Demographic Research*, 30, 253–276.

[ref57] Zaidi, B., & Morgan, S. P. (2017). The second demographic transition theory: A review and appraisal. *Annual Review of Sociology*, 43, 473–492. 10.1146/annurev-soc-060116-053442 PMC5548437 28798523

