# Supplementary material for: Two universal pathways in demographic transition
Source: Evol Hum Sci. 2026 Jun 15;8:e28. doi: 10.1017/ehs.2026.10054 (PMC13359015; doi:10.1017/ehs.2026.10054)
Supplement: Itao supplementary material 1 — Itao supplementary material [file S2513843X26100541sup001.pdf]

Supplementary Information for Two universal pathways in demographic transition

Kenji Itao[1,2,*]

[1] *Frontier Research Institute for Interdisciplinary Sciences, Tohoku University, Aramaki aza Aoba 6-3, Aoba-ku, Sendai 980-8578, Japan*
[2] *Center for Brain Science, RIKEN, 2-1 Hirosawa, Wako, Saitama 351-0198, Japan*
[*] *Corresponding author: kenji.itao.c6@tohoku.ac.jp*

SUPPLEMENTARY FIGURE

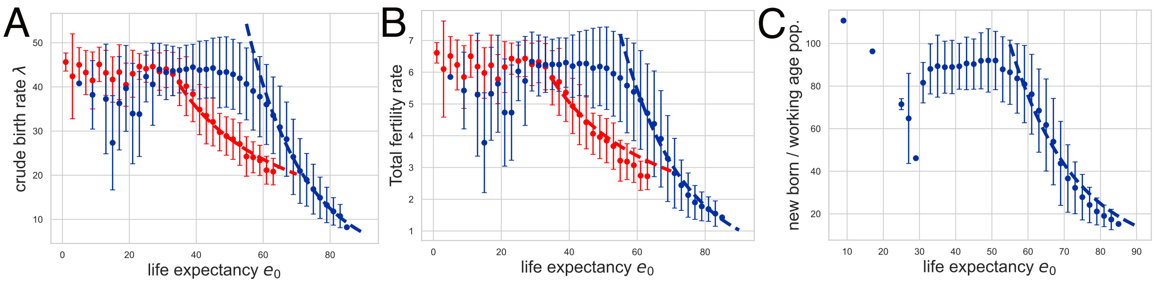

Figure S1. Robustness of the two-phase structure across fertility measures in the Gapminder data. Panels show the relationships between e_0 and (A) the crude birth rate λ, (B) the total fertility rate (TFR), and (C) the rescaled fertility rate, defined as the crude birth rate λ divided by the working-age (15–60) population ratio. Because working-age population data are available only after 1950, panel C shows results for Phase Ⅱ only. The threshold year is set to 1950, following the results in the main text.

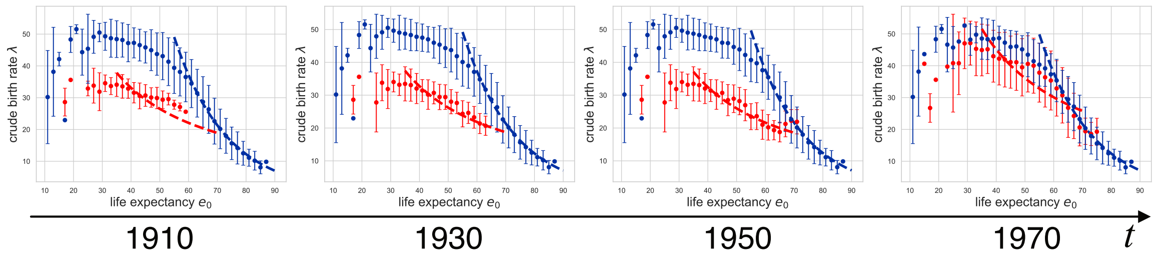

Figure S2. Dependence on the threshold year. Panels show the relationship between the crude birth rate λ and life expectancy e_0 before t (red) and after t (blue) for different threshold years t. The results remain consistent when t is set to 1930. However, when $t = 1910$, the fertility decline before t appears less pronounced, whereas when $t = 1970$, the λ–e_0 relationships overlap before and after t.

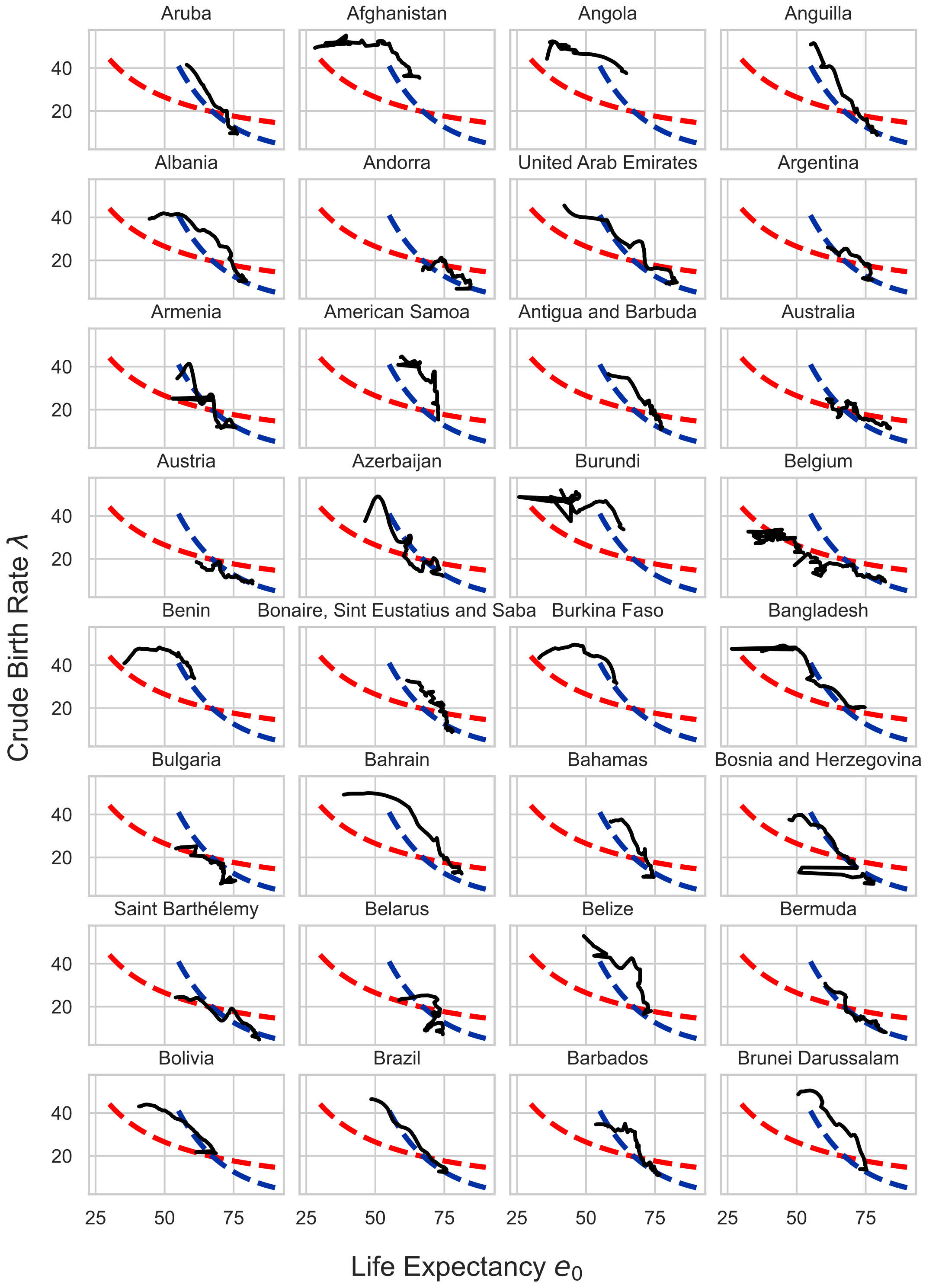

Figure S3. Pathways of countries, with two universal pathways as dashed lines.

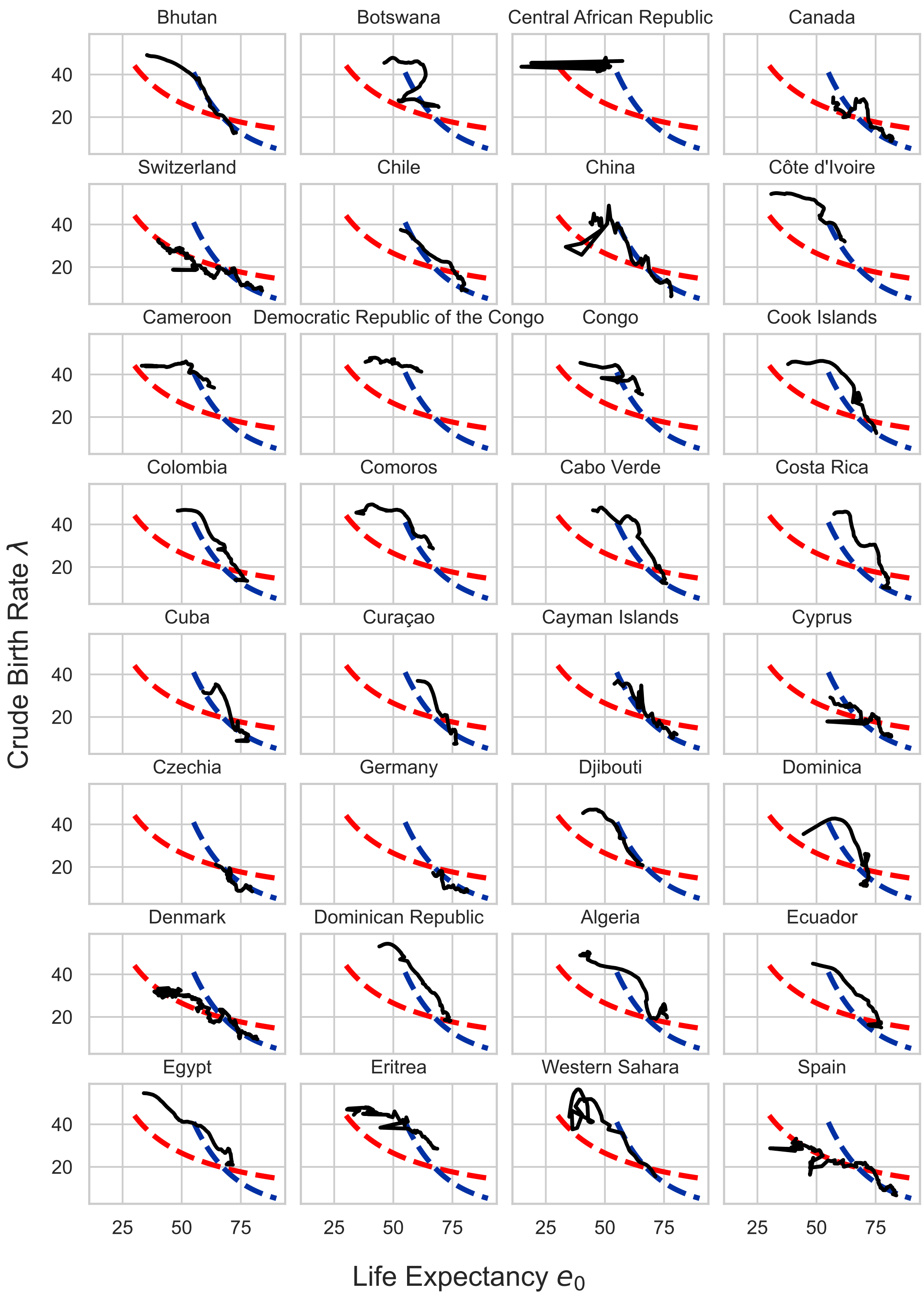

Figure S4. Pathways of countries, with two universal pathways as dashed lines.

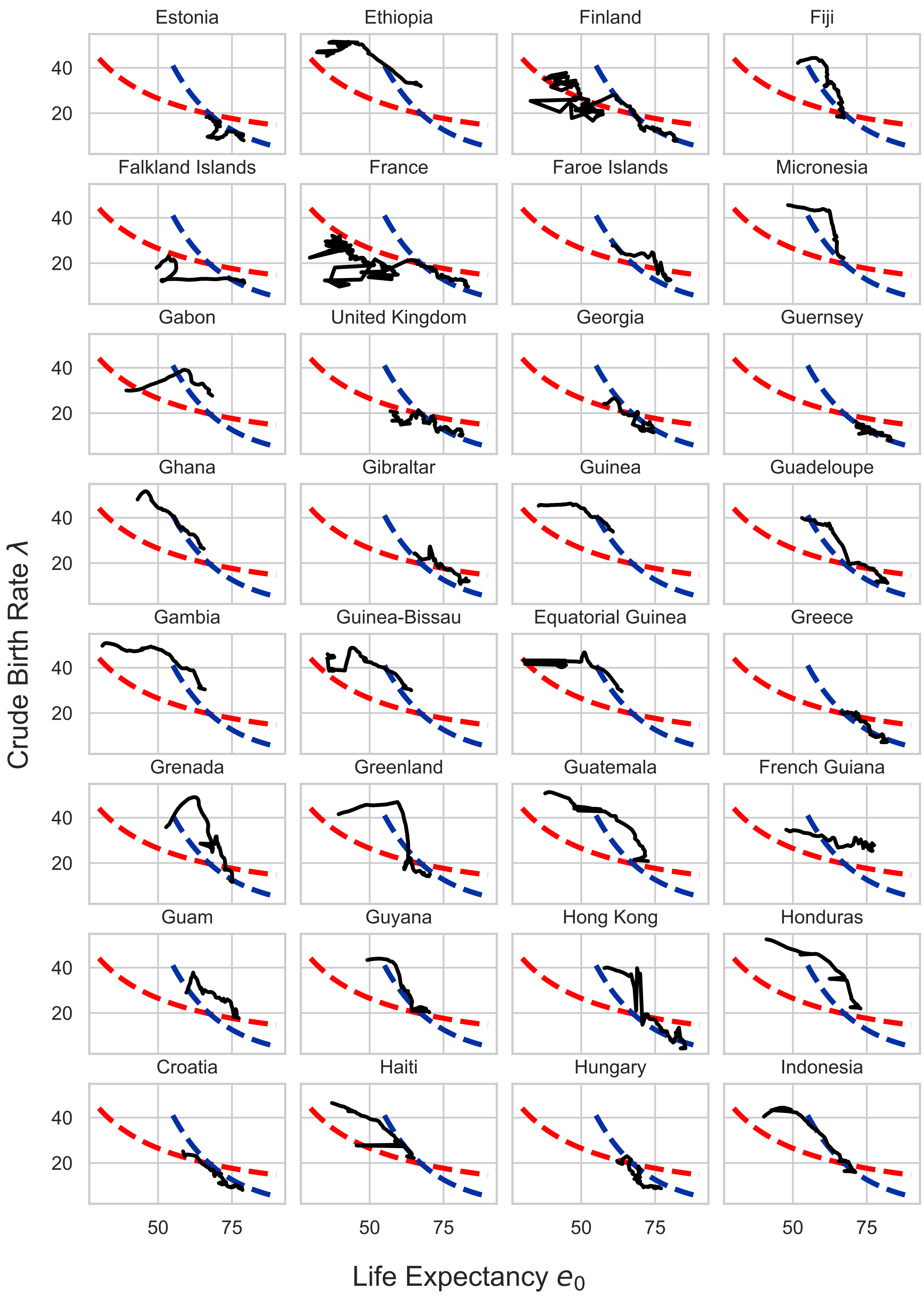

Figure S5. Pathways of countries, with two universal pathways as dashed lines.

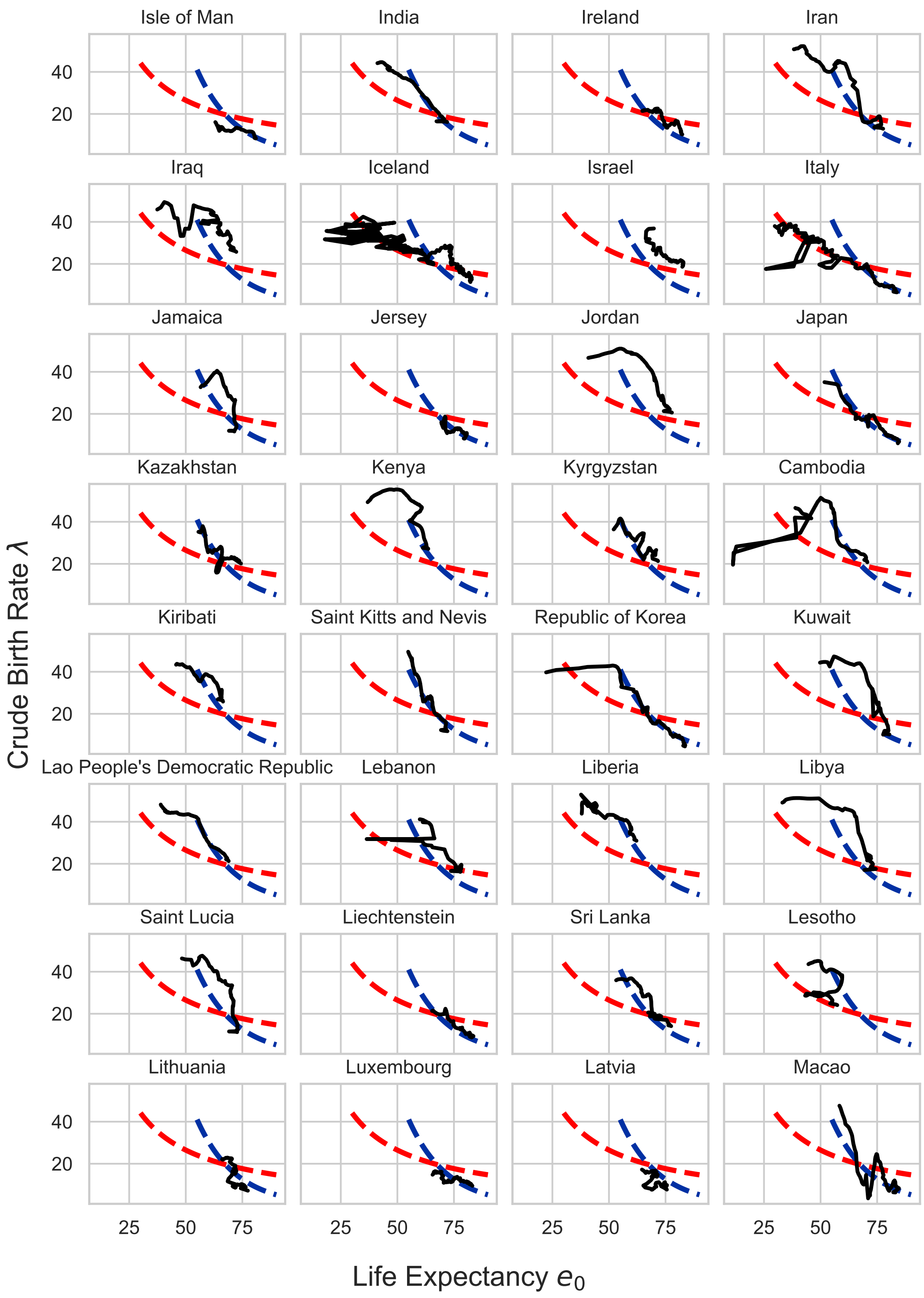

Figure S6. Pathways of countries, with two universal pathways as dashed lines.

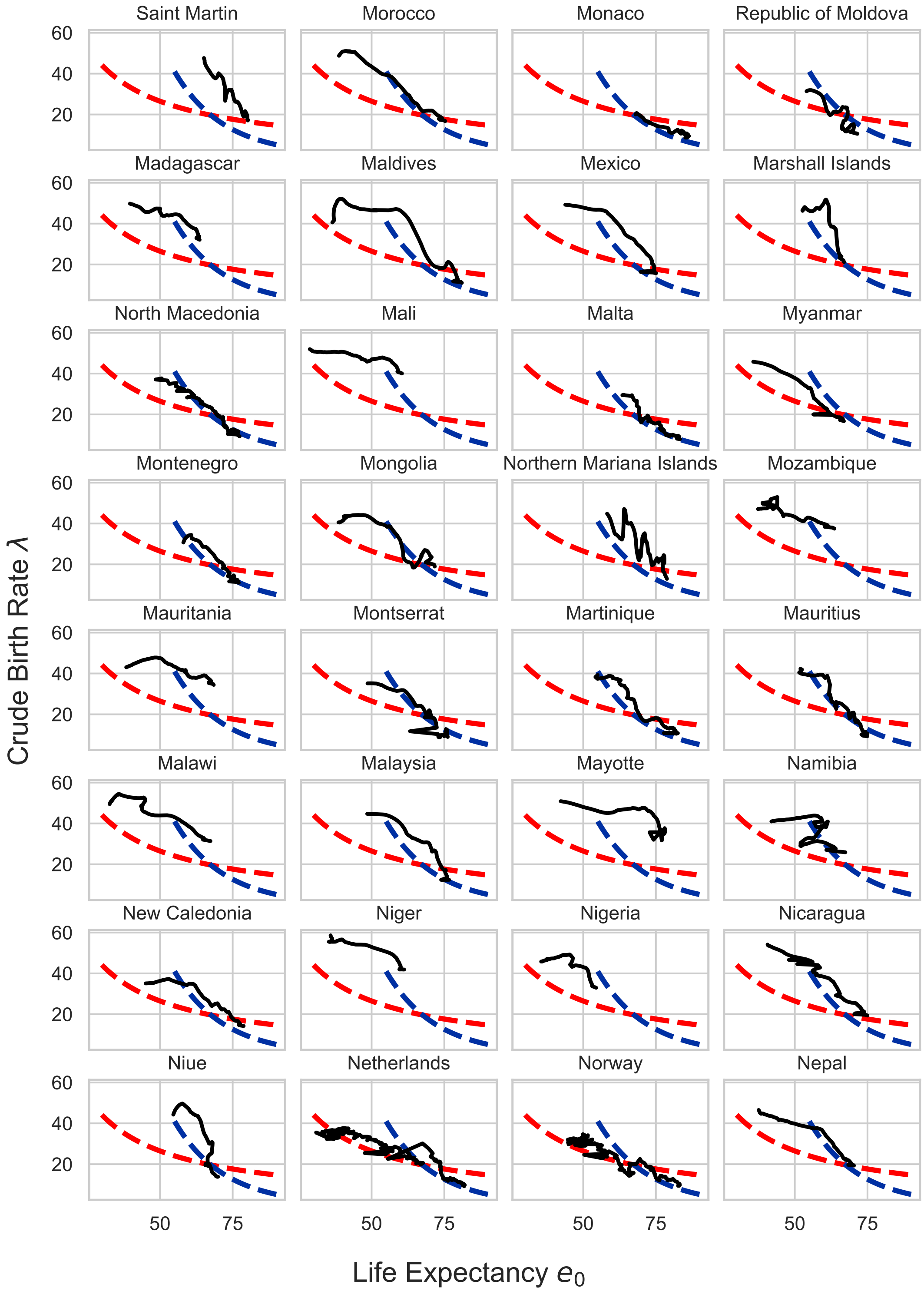

Figure S7. Pathways of countries, with two universal pathways as dashed lines.

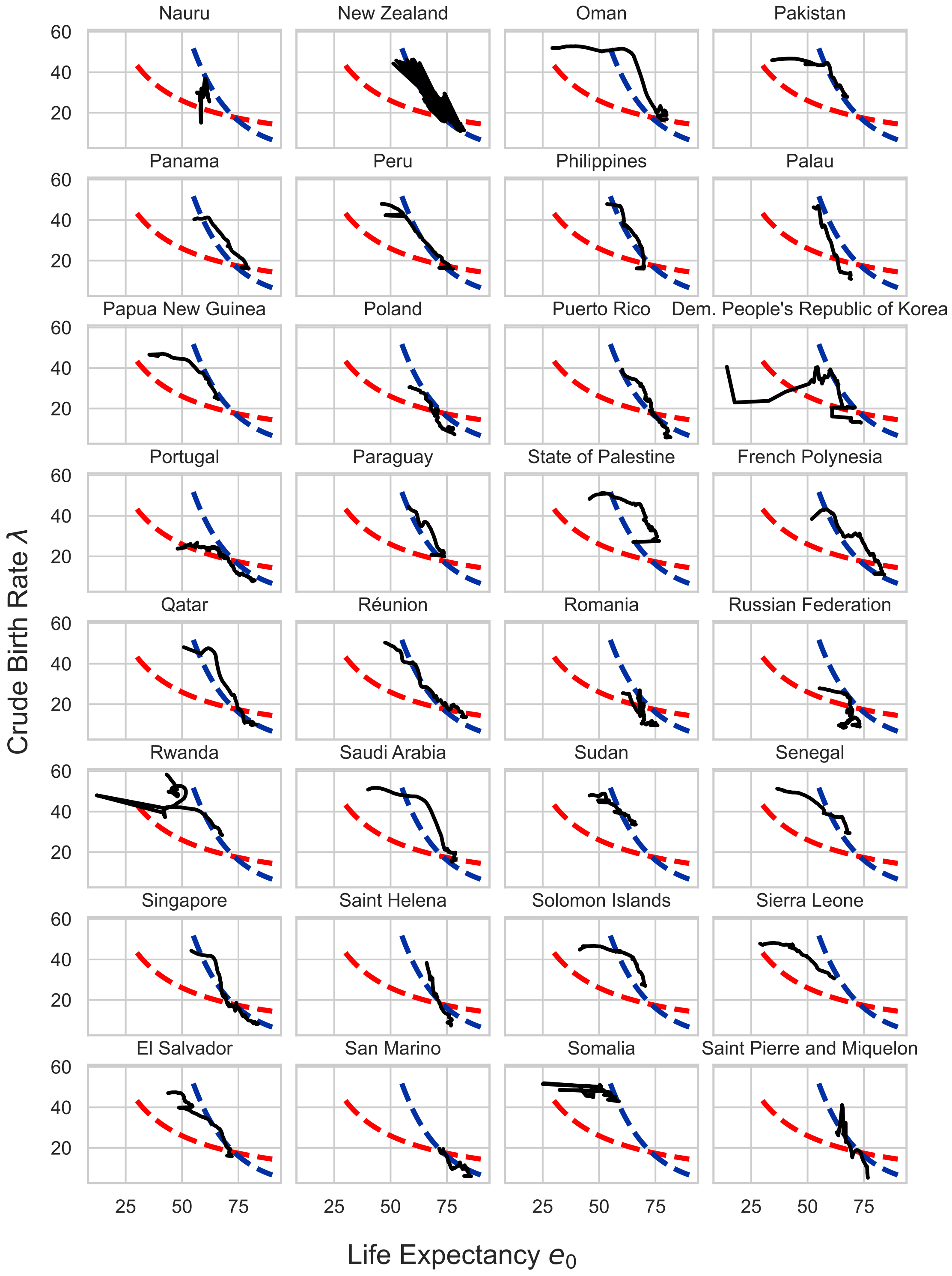

Figure S8. Pathways of countries, with two universal pathways as dashed lines.

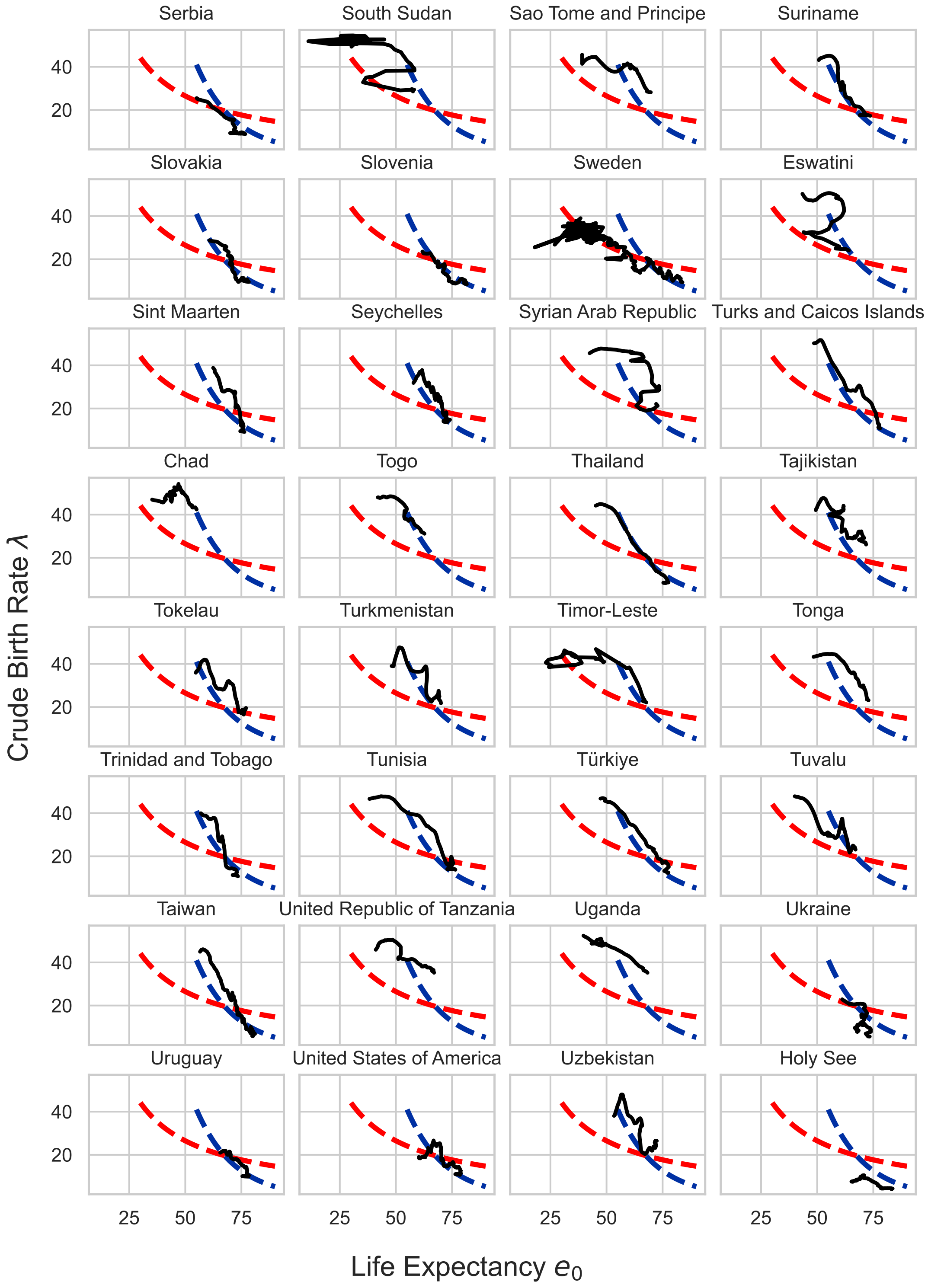

Figure S9. Pathways of countries, with two universal pathways as dashed lines.

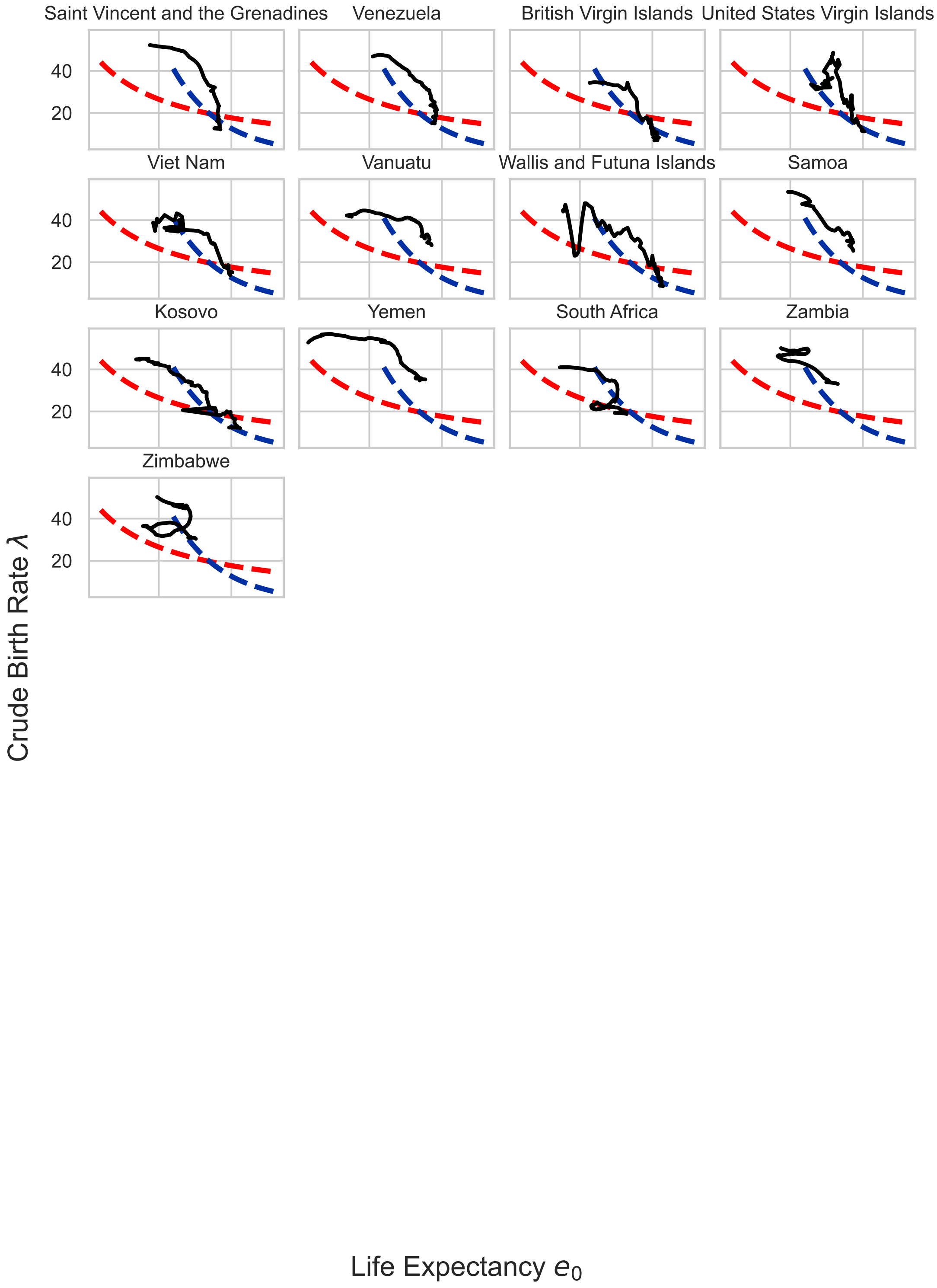

Figure S10. Pathways of countries, with two universal pathways as dashed lines.

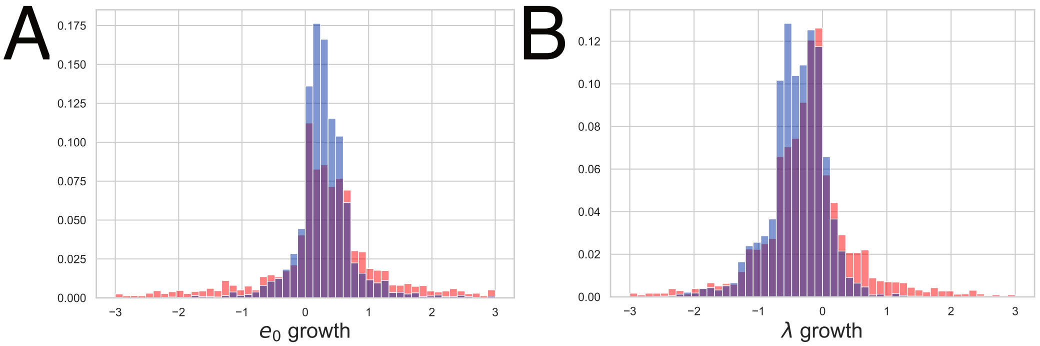

Figure S11. Indices characterising the two phases of demographic transition. The histograms show the annual changes in (A) life expectancy at birth e_0 and (B) the crude birth rate λ.

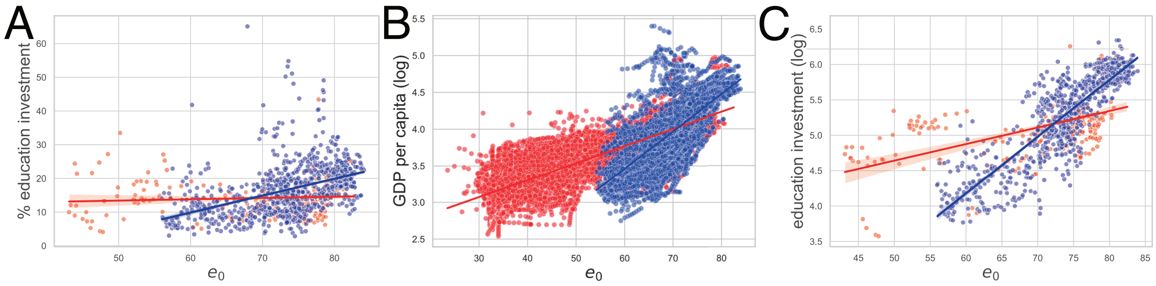

Figure S12. Educational investments across the two phases of demographic transitions. The scatterplots show (A) education expenditure per student relative to GDP per capita, (B) GDP per capita, and (C) education investment per student (log scale, USD), plotted against e_0. The y-axes in (B) and (C) are on a log scale. Panel (A) is identical to Fig. 3(D). Red and blue show the data of Phases I and Phase Ⅱ, respectively. Note that many countries shift from Phase I to Phase Ⅱ around $e_0 = 70$, indicating that educational investment increases much more sharply after this shift. The correlation between the per cent expenditure on education and e_0 (A) is 0.08 for Phase I and 0.43 for Phase Ⅱ. That between the logarithm of education investment per student and e_0 (C) is 0.56 for Phase I and 0.86 for Phase Ⅱ. Here, I use the Gapminder data because it provides broader coverage.

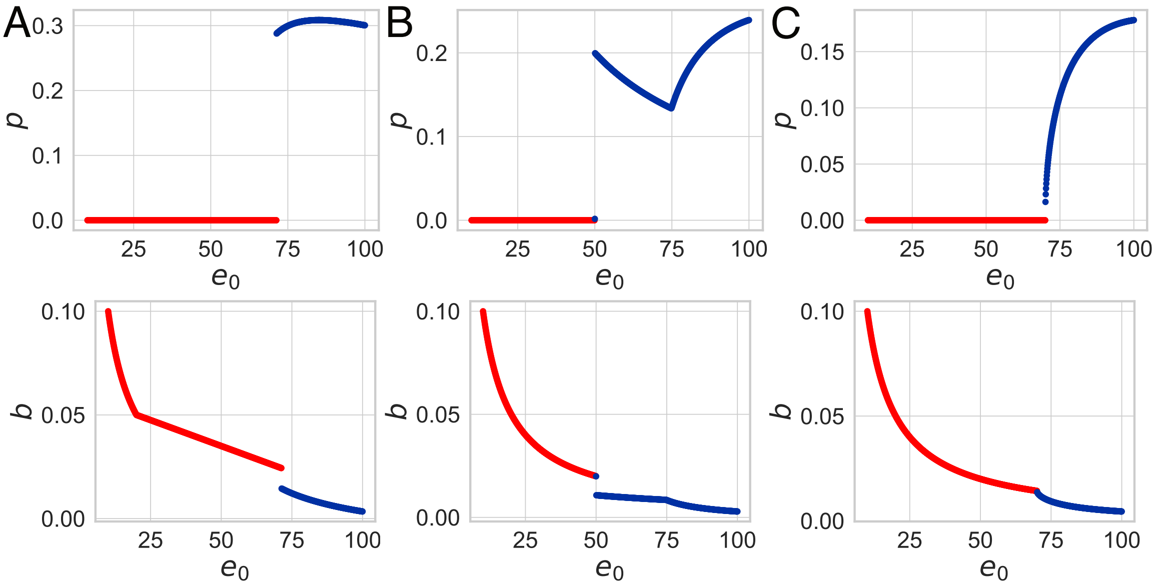

Figure S13. Numerical results for model variants. In each panel, the optimal fraction of lifetime allocated to education, p, and the optimal fertility rate, b, are plotted as functions of life expectancy at birth, e_0. (A) Variant in which parents pay each child's living costs only up to age 20, while adults pay their own living costs thereafter. (B) Variant in which children cannot engage in productive activities before age 10. (C) Variant in which older adults cannot engage in productive activities after age 70. The parameters are set to $\alpha = 0.2$, $\beta = 0.1$, and $c = 2$ in (A), and to $\alpha = 0.5$, $\beta = 0.1$, and $c = 25$ in (B) and (C). Points for Phase I ($p = 0$) are plotted in red, while those for Phase Ⅱ ($p > 0$) are plotted in blue.

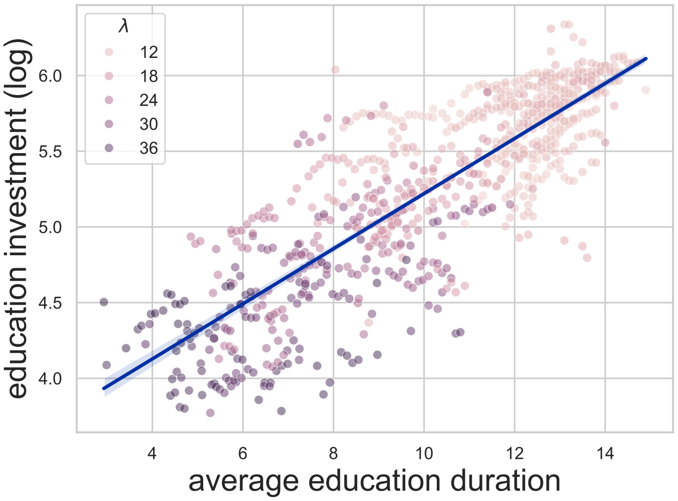

Figure S14. Relationship between average educational duration and education investment per student (log scale, USD). Colours represent the crude birth rate λ. The blue line denotes the results of linear regression. Data points below the blue line, representing relatively lower educational costs (c), are associated with higher fertility rates, as indicated by the darker colours. Here, I use the Gapminder data because it provides broader coverage.

SUPPLEMENTARY TABLE

Table S1. The year in which each country experienced each phase of demographic transition.

Phase I	Country	Phase II
—	Afghanistan	2011-2020
—	Albania	1961-2020
1997-2000	Algeria	1983-1996, 2001-2020
—	American Samoa	1967-2020
1950-1958, 1967-1976	Andorra	1959-1966, 1977-2020
—	Angola	2017-2020
—	Anguilla	1960-2020
1991-2002	Antigua and Barbuda	1950-1990, 2003-2020
1962-1969, 1999-2016	Argentina	1952-1961, 1970-1998, 2017-2020
1968-1977, 1993-1996	Armenia	1950-1967, 1978-1992, 1997-2020
—	Aruba	1951-2020
1921-1944	Australia	1945-2020
1947-1970	Austria	1971-2020
1972-1976, 1992-2011	Azerbaijan	1967-1971, 1977-1991, 2012-2020
—	Bahamas	1950-2020
1998-2016	Bahrain	1969-1997, 2017-2020
—	Bangladesh	1987-2020
—	Barbados	1950-2020
1950-1955	Belarus	1956-2020
1841-1845, 1850-1913, 1919-1966	Belgium	1967-2020
—	Belize	1964-1974, 1982-2020
—	Benin	2008-2020
1966-1974	Bermuda	1950-1965, 1975-2020
2000-2015	Bhutan	1990-1999, 2016-2020
—	Bolivia	1986-2020
1971-1977, 1988-1996	Bonaire, Sint Eustatius and Saba	1950-1970, 1978-1987, 1997-2020
1969-1988	Bosnia and Herzegovina	1956-1968, 1989-2020
1995-2009	Botswana	1984-1994, 2010-2020
2000-2005	Brazil	1966-1999, 2006-2020
1983-1986	British Virgin Islands	1950-1982, 1987-2020
1999-2004	Brunei Darussalam	1963-1998, 2005-2020
1947-1977	Bulgaria	1978-2020
—	Burkina Faso	2015-2020
—	Burundi	2016-2020
—	Cabo Verde	1989-2020

Phase I	Country	Phase Ⅱ
—	Cambodia	1995-2020
—	Cameroon	2010-2020
1921-1942	Canada	1943-2020
1975-1985	Cayman Islands	1950-1974, 1986-2020
1992-2001	Chile	1952-1991, 2002-2020
1975-1984, 1991-1995	China	1967-1974, 1985-1990, 1996-2020
2006-2009	Colombia	1967-2005, 2010-2020
—	Comoros	1994-2020
—	Congo	1984-2020
—	Cook Islands	1967-2020
1993-2015	Costa Rica	1965-1992, 2016-2020
1950-1974	Croatia	1975-2020
—	Cuba	1950-2020
—	Curaçao	1950-2020
1950-1964, 1973-1994	Cyprus	1965-1972, 1995-2020
1950-1980	Czechia	1981-2020
—	Côte d'Ivoire	2014-2020
1953-1956, 1976-1985, 1995-2008	Dem. People's Republic of Korea	1959-1975, 1986-1994, 2009-2020
1836-1943, 1949-1955	Denmark	1944-1948, 1956-2020
2008-2020	Djibouti	1989-2007
—	Dominica	1963-2020
—	Dominican Republic	1972-2020
2010-2019	Ecuador	1971-2009
—	Egypt	1981-2020
2013-2020	El Salvador	1983-2012
—	Equatorial Guinea	2006-2020
—	Eritrea	2000-2020
1950-1964, 1969-1990	Estonia	1965-1968, 1991-2020
1996-2020	Eswatini	1990-1995
—	Ethiopia	2008-2020
1950-1998	Falkland Islands	1999-2020
1970-1981, 1986-1993	Faroe Islands	1950-1969, 1982-1985, 1994-2020
2011-2020	Fiji	1965-2010
1882-1945, 1951-1969	Finland	1946-1950, 1970-2020
1836-1969	France	1970-2020
1950-1958	French Guiana	1959-2020
1995-2016	French Polynesia	1966-1994, 2017-2020
1952-1969	Gabon	1970-2020

Phase I	Country	Phase Ⅱ
—	Gambia	2002-2020
1950-1995	Georgia	1996-2020
1950-1968	Germany	1969-2020
—	Ghana	1992-2020
1974-2000	Gibraltar	1950-1973, 2001-2020
1950-1964	Greece	1965-2020
1970-2020	Greenland	1966-1969
—	Grenada	1966-2020
1988-2006	Guadeloupe	1954-1987, 2007-2020
2001-2020	Guam	1950-2000
—	Guatemala	1986-2020
—	Guernsey	1951-2020
—	Guinea	2010-2020
—	Guinea-Bissau	2009-2020
2002-2012	Guyana	1964-2001, 2013-2020
—	Haiti	1992-2018
1950-1953	Holy See	1954-2020
—	Honduras	1986-2020
—	Hong Kong	1950-2020
1950-1981	Hungary	1982-2020
1841-1848, 1876-1941, 1970-2014	Iceland	1942-1969, 2015-2020
2008-2020	India	1981-2007
1999-2020	Indonesia	1973-1998
1995-2003, 2011-2018	Iran	1986-1994, 2004-2010
—	Iraq	1979-2020
2006-2014	Ireland	1952-2005, 2015-2020
1950-1981	Isle of Man	1982-2020
1987-2020	Israel	1950-1986
1896-1970	Italy	1971-2020
2004-2008	Jamaica	1950-2003, 2009-2020
1947-1965	Japan	1966-2020
1950-1953, 1959-1967	Jersey	1954-1958, 1968-2020
2017-2020	Jordan	1985-2016
1966-1979, 1991-2007	Kazakhstan	1950-1965, 1980-1990, 2008-2020
—	Kenya	2002-2020
—	Kiribati	1971-2020
2006-2011	Kosovo	1971-2005, 2012-2020
1998-2015	Kuwait	1973-1997, 2016-2020

Phase I	Country	Phase Ⅱ
1997-2004	Kyrgyzstan	1964-1996, 2005-2020
—	Lao People’s Democratic Republic	1995-2020
1950-1963, 1970-1995	Latvia	1964-1969, 1996-2020
2001-2020	Lebanon	1952-2000
1995-2020	Lesotho	1981-1994
—	Liberia	2007-2020
—	Libya	1983-2020
1950-1958	Liechtenstein	1959-2020
1950-1955, 1965-1972	Lithuania	1956-1964, 1973-2020
1950-1967	Luxembourg	1968-2020
1964-1969, 1988-1993	Macao	1955-1963, 1970-1987, 1994-2020
—	Madagascar	2002-2020
—	Malawi	2011-2020
2003-2012	Malaysia	1963-2002, 2013-2020
2003-2016	Maldives	1991-2002, 2017-2020
—	Mali	—
1964-1974	Malta	1950-1963, 1975-2020
—	Marshall Islands	1992-2020
1975-1979, 1984-1993	Martinique	1952-1974, 1980-1983, 1994-2020
—	Mauritania	1992-2020
1984-1989, 1994-1999	Mauritius	1957-1983, 1990-1993
—	Mayotte	1997-2020
2011-2020	Mexico	1975-2010
—	Micronesia	1982-2020
1950-1959	Monaco	1960-2020
1993-2007	Mongolia	1980-1992, 2008-2020
1978-1984	Montenegro	1950-1977, 1985-2020
1950-1954, 1977-1981, 1987-1993	Montserrat	1955-1976, 1982-1986, 1994-2020
2016-2020	Morocco	1981-2015
—	Mozambique	2015-2020
1986-2020	Myanmar	1975-1985
—	New Zealand	1948-2020
1999-2010	Namibia	1979-1998, 2011-2020
1950-1979, 2001-2004	Nauru	1980-2000, 2005-2020
—	Nepal	1990-2019
1874-1945, 1965-1970	Netherlands	1946-1964, 1971-2020
1950-1955, 2001-2014	New Caledonia	1962-2000, 2015-2020
—	Nicaragua	1986-2020

Phase I	Country	Phase II
—	Nigeria	2018-2020
1995-2020	Niue	1970-1994
1981-1994	North Macedonia	1953-1980, 1995-2020
2002-2020	Northern Mariana Islands	1955-2001
1846-1945, 1953-1957, 1965-1968	Norway	1946-1952, 1958-1964, 1969-2020
2004-2020	Oman	1988-2003
—	Pakistan	1994-2020
1978-2017	Palau	1965-1977, 2018-2020
2005-2020	Panama	1957-2004
—	Papua New Guinea	1978-2020
—	Paraguay	1965-2020
2012-2020	Peru	1974-2011
2018-2020	Philippines	1969-2017
1961-1987	Poland	1950-1960, 1988-2020
1940-1961, 1968-1978	Portugal	1962-1967, 1979-2020
—	Puerto Rico	1950-2020
1994-2004	Qatar	1971-1993, 2005-2020
1975-1989	Republic of Korea	1963-1974, 1990-2020
1962-1982, 1989-2016	Republic of Moldova	1953-1961, 1983-1988, 2017-2020
1950-1990	Romania	1991-2020
1950-1954, 1962-2010	Russian Federation	1955-1961, 2011-2020
—	Rwanda	2006-2020
1988-2020	Réunion	1961-1987
1950-1976, 1983-1990	Saint Barthélemy	1977-1982, 1991-2020
—	Saint Helena	1950-2020
1991-2005	Saint Kitts and Nevis	1962-1990, 2006-2020
—	Saint Lucia	1965-2020
2001-2020	Saint Martin	1958-2000
1973-1991	Saint Pierre and Miquelon	1950-1972, 1992-2020
1999-2015	Saint Vincent and the Grenadines	1971-1998, 2016-2020
—	Samoa	1971-2020
—	San Marino	1950-2020
—	Sao Tome and Principe	1968-1975, 1985-2020
2005-2020	Saudi Arabia	1988-2004
—	Senegal	1993-2020
1950-1973	Serbia	1974-2020
1997-2003	Seychelles	1950-1996, 2004-2020
—	Sierra Leone	2013-2020

Phase I	Country	Phase II
1974-1982	Singapore	1958-1973, 1983-2020
—	Sint Maarten	1950-2020
1965-1972, 1980-1988	Slovakia	1950-1964, 1973-1979, 1989-2020
1950-1979	Slovenia	1980-2020
—	Solomon Islands	1984-2020
1994-2020	South Africa	1971-1993
2018-2020	South Sudan	2010-2013
1908-1957	Spain	1958-2020
1995-2000	Sri Lanka	1950-1994, 2001-2020
—	State of Palestine	1996-2020
—	Sudan	—
2015-2020	Suriname	1965-2014
1800-1805, 1815-1950	Sweden	1951-2020
1876-1962	Switzerland	1963-2020
2015-2018	Syrian Arab Republic	1986-2014
—	Taiwan	1959-2020
—	Tajikistan	1978-1983, 1994-2020
1982-1998	Thailand	1969-1981, 1999-2020
—	Timor-Leste	2000-2020
—	Togo	2000-2020
2009-2020	Tokelau	1950-1955, 1963-2008
—	Tonga	1967-2020
1990-2005	Trinidad and Tobago	1950-1989, 2006-2020
2010-2019	Tunisia	1973-2009
—	Turkmenistan	1969-2020
2002-2007	Turks and Caicos Islands	1962-2001, 2008-2020
1963-1975, 2003-2014	Tuvalu	1976-2002, 2015-2020
2004-2018	Türkiye	1971-2003
—	Uganda	2013-2020
1950-1958, 1975-1985	Ukraine	1959-1974, 1986-2020
2000-2005	United Arab Emirates	1965-1999, 2006-2020
1922-1966	United Kingdom	1967-2020
—	United Republic of Tanzania	2007-2020
—	United States Virgin Islands	1950-1963, 1972-2020
1933-1945, 1965-1971	United States of America	1946-1964, 1972-2020
1950-1957, 1965-1972, 1979-1987	Uruguay	1958-1964, 1973-1978, 1988-2020
1999-2006	Uzbekistan	1967-1998, 2007-2020
—	Vanuatu	1976-2020

Phase I	Country	Phase Ⅱ
—	Venezuela	1966-2020
2012-2015	Viet Nam	1961-2011, 2016-2020
1959-1966, 2000-2003	Wallis and Futuna Islands	1976-1999, 2004-2020
1999-2020	Western Sahara	1990-1998
—	Yemen	2000-2020
—	Zambia	2012-2020
1993-2007	Zimbabwe	1986-1992, 2012-2020
