# Supplementary material for: Two universal pathways in demographic transition
Source: Evol Hum Sci. 2026 Jun 15;8:e28. doi: 10.1017/ehs.2026.10054 (PMC13359015; doi:10.1017/ehs.2026.10054)
Supplement: Itao supplementary material 2 — Itao supplementary material [file S2513843X26100541sup002.rtf]

Global data reveal two universal pathways linking fertility and longevity; countries follow or switch between them.
